# A Nonanuclear Pd-Based Coordination Cage Featuring Ni-Chelated Porphyrin Ligand for Cancer Therapy via Combination of Chemodynamic and Photodynamic Modalities

**DOI:** 10.3390/molecules31111874

**Published:** 2026-05-29

**Authors:** Meng-Lin Dong, Ye Ning, Yuan-Hui Jia, Wen-Hua Zhang, Wenqiang Lu, Yiming Mao

**Affiliations:** 1 College of Chemistry, Chemical Engineering and Materials Science, Soochow University, Suzhou 215123, China; 20234209141@stu.suda.edu.cn (M.-L.D.); 15854833290@163.com (Y.N.); yh1204329265@163.com (Y.-H.J.); 2 Department of Thoracic Surgery, Suzhou Kowloon Hospital, Shanghai Jiao Tong University School of Medicine, Suzhou 215021, China

**Keywords:** coordination cage, reactive oxygen species, photodynamic therapy, chemodynamic therapy, anticancer drugs

## Abstract

Reactive oxygen species (ROS)-mediated cancer therapy has attracted extensive attention due to its high spatiotemporal selectivity and minimal side effects. Herein, we report a nonanuclear Pd-based coordination cage of **Pd_6_-TMPP(Ni)**, constructed from Ni-chelated TMPP(Ni) as the metalloligand and Pd^2+^ ions (**H_2_TMPP** = *meso*-tetrakis (6-methylpyridin-3-yl) porphyrin). **Pd_6_-TMPP(Ni)** integrates dual ROS-generation for cancer therapy, viz., hydroxyl radical (•OH) production and photoinduced singlet oxygen (^1^O_2_) generation. In vitro cytotoxicity assays against cancer cell lines NCI-H82 (lung cancer), A549 (lung cancer), KYSE-510 (esophageal cancer), and Te-1 (esophageal cancer) reveal its potent dark toxicity (IC_50_: 1.9–2.1 μmol L^−1^) and phototoxicity (IC_50_: 0.8–1.5 μmol L^−1^), which is attributed to enhanced intracellular ROS accumulation. This work develops a versatile therapeutic platform that harnesses Ni-induced •OH for chemodynamic therapy (CDT) and porphyrin-generated ^1^O_2_ for photodynamic therapy (PDT), thereby mitigating the oxygen dependence of conventional PDT.

## 1. Introduction

Reactive oxygen species (ROS), particularly hydroxyl radical (•OH), singlet oxygen (^1^O_2_), and hydrogen peroxide (H_2_O_2_), have emerged as promising agents for cancer therapy due to their ability to induce oxidative stress and disrupt cellular homeostasis, offering distinct advantages in tumor selectivity and the potential to overcome conventional drug resistance [1,2]. Among various ROS-generating strategies, transition metal-catalyzed Fenton or Fenton-like reactions have garnered significant attention due to their ability to convert H_2_O_2_ into cytotoxic •OH [3,4,5]. Nickel complexes have demonstrated remarkable catalytic activity in this context [6,7,8]. The ability of Ni(II) to undergo reversible redox cycling with Ni(III) enables efficient decomposition of H_2_O_2_ to generate •OH radicals, a mechanism that has been extensively exploited in environmental remediation for the degradation of tough organic pollutants [7].

In the context of ROS-based cancer therapies, Ni(II)-induced •OH generation also offers an advantage as it can be produced by consumption of endogenously overexpressed H_2_O_2_ (typically 10–50 μM) in the tumor microenvironment (TME) [9,10]. This TME-dependent ROS production circumvents the O_2_ limitation and penetration depth limitations associated with conventional O_2_-dependent therapies such as type II photodynamic therapy (PDT) [11,12,13]. For example, NiCl_2_ has long been documented to induce lipid peroxidation and DNA damage via the Fenton-like process [14]. Liu et al. recently reported that nickel-based single-atom-metal-clusters (NSAMCs) exhibit peroxidase-like activity, catalyzing H_2_O_2_ in the TME to generate •OH, which synergizes with glutathione (GSH) depletion to boost ferroptosis [15].

Complementary to Ni-induced •OH production, photoinduced ROS generation, and ^1^O_2_ in particular, have become a cornerstone of PDT, an approach that relies on photosensitizers activated by specific light wavelengths [16,17]. Porphyrins and their derivatives are among the most widely used photosensitizers due to their strong absorption in the visible-near-infrared region and high ^1^O_2_ quantum yields [18,19]. However, their application is hindered by inherent limitations, including self-aggregation resulting from π–π stacking, poor water solubility, and low tumor targeting efficiency [20]. To address these challenges, researchers have integrated porphyrins into metal–organic frameworks or discrete coordination cages via coordination with metal ions [21,22,23,24]. These hybrid structures not only inhibit porphyrin stacking to preserve photosensitizer activity but also enhance ROS generation efficacy by optimizing light absorption, improving cellular uptake, and enabling controlled release of ROS in the TME [25,26,27].

**Scheme 1:**
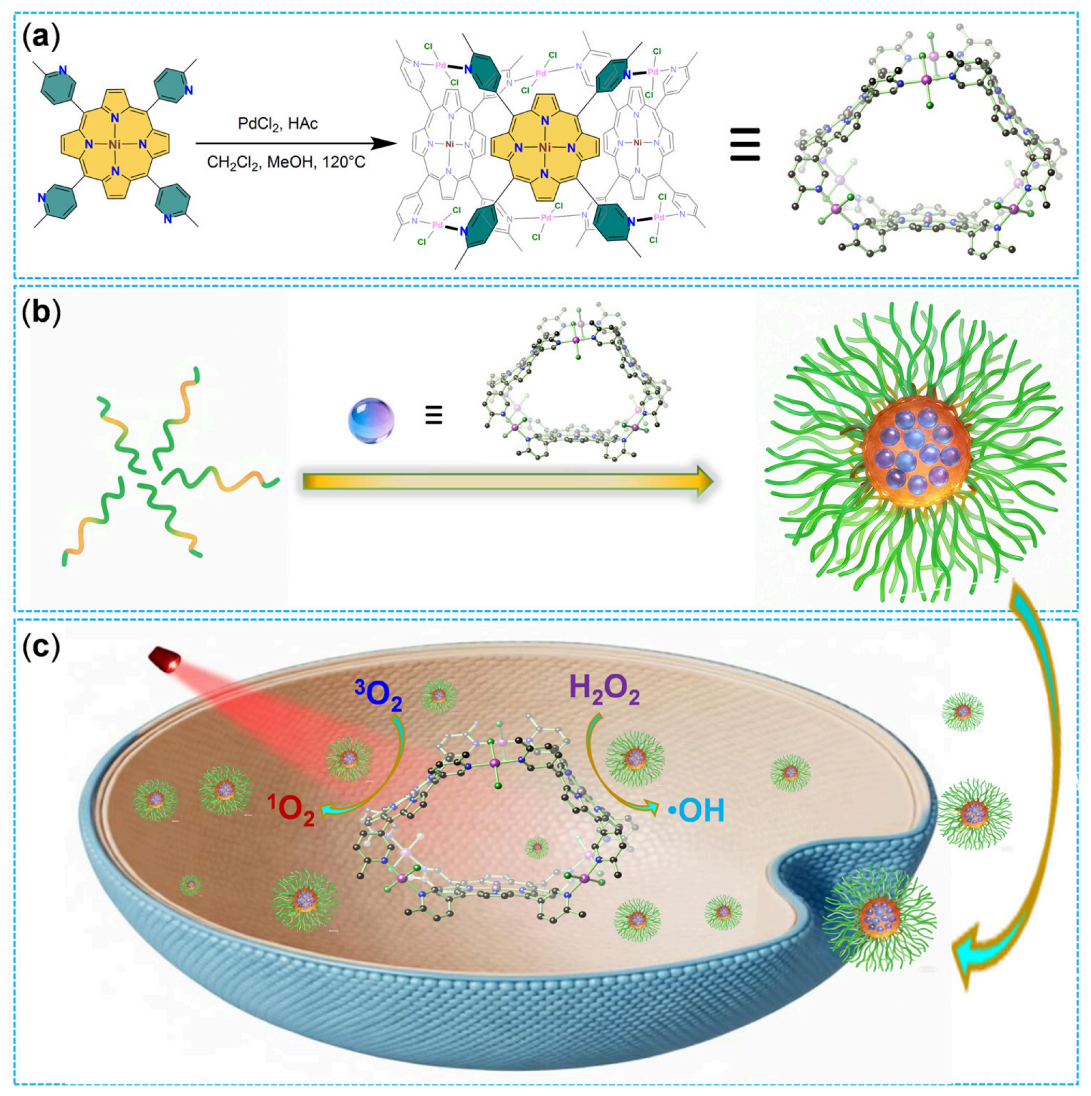
The material synthesis, particle assembly, and anticancer mechanism. (**a**) The synthetic procedure for **Pd_6_-TMPP(Ni)**, its assembly with (**b**) Pluronic F127 to give **Pd_6_-TMPP(Ni)-F127**, and (**c**) an illustration of the anticancer mechanism of **Pd_6_-TMPP(Ni)-F127**.

**Figure 1:**
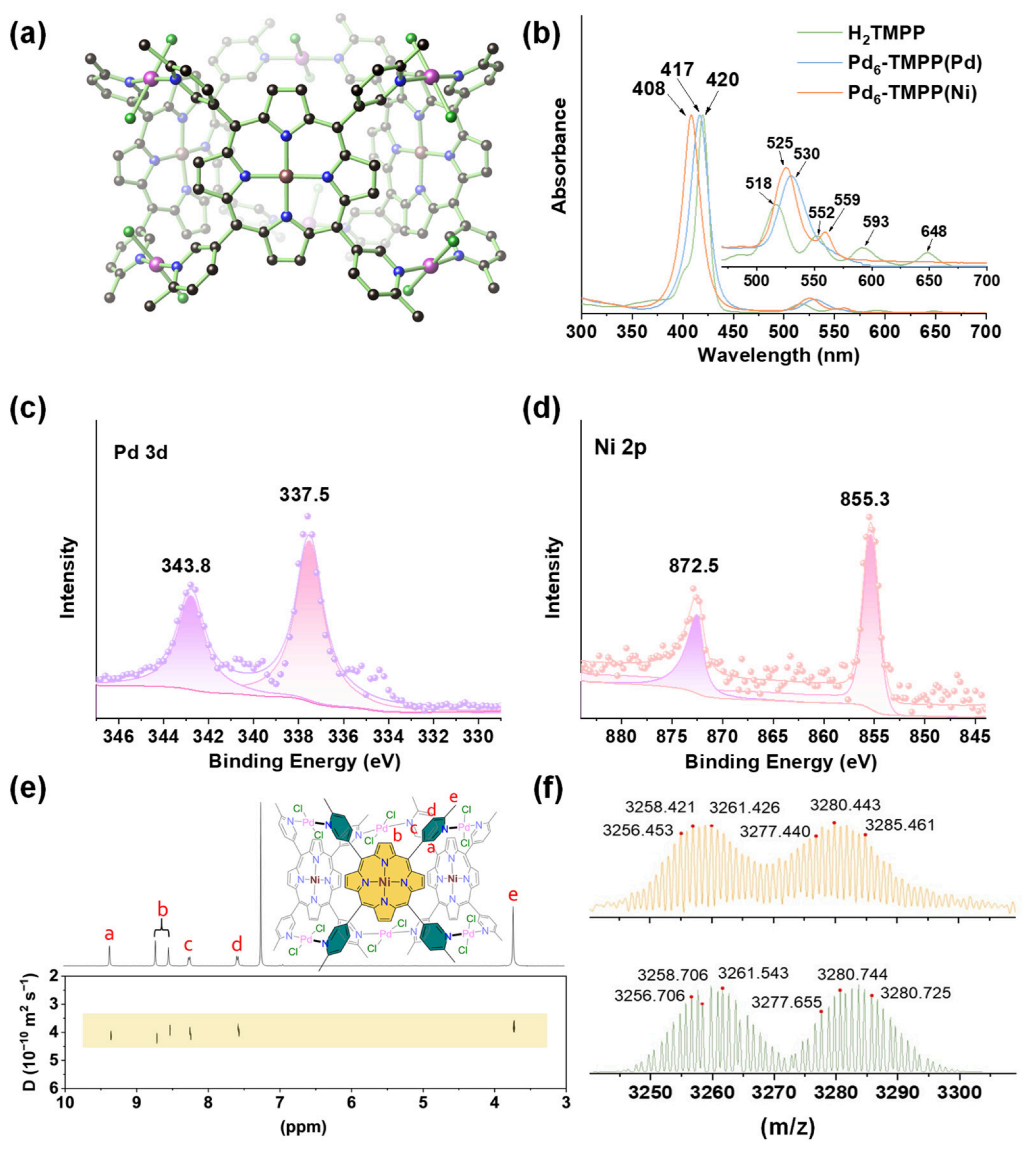
Spectroscopic and spectrometric characterizations of **Pd_6_-TMPP(Ni)**. (**a**) The X-ray structure of **Pd_6_-TMPP(Ni)**. (**b**) The UV-Vis spectra of **H_2_TMPP**, **Pd_6_-TMPP(Pd)**, and **Pd_6_-TMPP(Ni)** (inset: the amplified absorption in the range of 475–700 nm). (**c**) Pd 3d_5/2, 3/2_ and (**d**) Ni 2p_3/2, 1/2_ XPS spectra of **Pd_6_-TMPP(Ni)**. (**e**) The ^1^H DOSY spectrum of **Pd_6_-TMPP(Ni)** (298 K, CDCl_3_), and (**f**) the experimental (orange yellow) and calculated (light green) MALDI-TOF MS spectrometry of **Pd_6_-TMPP(Ni)**. For (**a**), the hydrogen atoms are omitted for clarity. Color legend: Pd (dark magenta), Ni (brown), Cl (green), N (blue), and C (black).

Herein, we report a coordination cage of **Pd_6_-TMPP(Ni)** constructed from Ni-chelated porphyrin metalloligand TMPP(Ni) and Pd^2+^ (**H_2_TMPP** = *meso*-tetrakis (6-methylpyridin-3-yl) porphyrin) (Scheme 1a). **Pd_6_-TMPP(Ni)** features a hexagonal prismatic structure wherein the three TMPP(Ni) metalloligands occupy alternative lateral faces of the prism, juxtaposing three pairs of PdCl_2_ units via binding to the *trans* positions of the Pd^2+^ ion (Scheme 1 and Figure 1a). The TMPP(Ni) moieties in **Pd_6_-TMPP(Ni)** serve as both a catalyst for •OH production via Fenton-like reactions and a photosensitizer for light-induced ^1^O_2_ generation (Scheme 1c). The CCK-8 assays indicated that **Pd_6_-TMPP(Ni)** is toxic for cell lines, including lung cancer NCI-H82 and A549, as well as esophageal cancer KYSE-510 and Te-1, featuring half maximal inhibitory concentration (IC_50_) values in the range of 1.9–2.1 μmol L^−1^, which can be further lowered to 0.8–1.5 μmol L^−1^ with the presence of 650 nm light irradiation. The cytotoxicity of **Pd_6_-TMPP(Ni)** outperforms that of the free ligand **H_2_TMPP** and the Pd congener of **Pd_6_-TMPP(Pd)**.

## 2. Results and Discussion

### 2.1. Synthesis and Structure of Pd_6_-TMPP(Ni)

The coordination cage of **Pd_6_-TMPP(Ni)** was obtained in a 16.4% yield as red needle-like crystals from the solvothermal reaction of TMPP(Ni) and PdCl_2_ in CH_2_Cl_2_/MeOH with HOAc as the modulator. **Pd_6_-TMPP(Ni)** is soluble in CH_2_Cl_2_, DMF, and DMSO, slightly soluble in MeOH and MeCN, but insoluble in H_2_O.

Single-crystal X-ray diffraction analysis revealed that **Pd_6_-TMPP(Ni)** crystallizes in the monoclinic *P*2_1_/*c* space group (Table 1). The asymmetric unit of **Pd_6_-TMPP(Ni)** contains a full molecule (C_1_ point group, Figure 1a and Scheme 1a) instead of the expected D_3h_ or C_2v_ symmetry as that found for **Pd_6_-TMPP(Au)** [28].

In **Pd_6_-TMPP(Ni)**, three pairs of PdCl_2_ units were juxtaposed by three TMPP(Ni) metalloligands to give a hexagonal prismatic structure. The side lengths of the two Pd-based triangles from the base planes, i.e., Pd1∙∙∙Pd2/Pd2∙∙∙Pd3/Pd1∙∙∙Pd3 being 12.2020(17)/11.9926(18)/13.5157(19) Å, and Pd4∙∙∙Pd5/Pd5∙∙∙Pd6/Pd4∙∙∙Pd6 being 12.1185(18)/11.7959(18)/13.611(2) Å (Table S1), are similar but both deviate from an equilateral triangle or an isosceles triangle. In addition, the triangle formed by three Ni ions, viz., Ni1∙∙∙Ni2/Ni2∙∙∙Ni3/Ni1∙∙∙Ni3, is 10.303(3)/10.665(3)/12.463(3) Å (Table S1).

It is notable that the small ionic radius for Ni^2+^ (0.63 Å) also caused the saddle-shaped ruffling of the three porphyrin backbones (the twelve Ni–N bond distances are in the range of 1.904(12)–1.983(12) Å, Table S1) [29], featuring six pyrrole rings pointing inwards to the centers of the two base planes to create a hydrophobic pocket that may be suitable for storing small molecules [30]. Platon void calculation also suggested that the guest-accessible void of **Pd_6_-TMPP(Ni)** was sufficiently large, occupying 43.6% (8369.2 Å^3^ of 19,210.0 Å^3^) of the total cell volume [31].

### 2.2. Nuclear Magnetic Resonance

To confirm the identity and purity of the as-synthesized **Pd_6_-TMPP(Ni)**, we performed ^1^H nuclear magnetic resonance (NMR) characterizations. As shown in Figure S1, the ^1^H NMR indicated that the characteristic peak of pyrrole protons at −2.81 ppm in **H_2_TMPP** disappeared after the formation of TMPP(Ni), which directly confirmed that Ni^2+^ had been successfully incorporated into the porphyrin center [18,28]. The diffusion ordered spectroscopy (DOSY) for **Pd_6_-TMPP(Ni)** shows a narrow diffusion band with diffusion coefficients of 3.83 × 10^−10^ m^2^ s^−1^ (Figure 1e), suggesting the formation of single assemblies.

### 2.3. Ultraviolet–Visible Absorption Spectra

In the ultraviolet–visible (UV-Vis) absorption spectrum (Figure 1b), the Soret band of **Pd_6_-TMPP(Ni)** blue-shifted to 408 nm as compared to those of **H_2_TMPP** (420 nm) and **Pd_6_-TMPP(Pd)** (417 nm) [32]. Notably, the four Q bands of **H_2_TMPP** in the range of 500–700 nm coalesced into two peaks (525/559 nm) in **Pd_6_-TMPP(Ni)** (Figure 1b, inset) due to the metalation that elevated the symmetry of the porphyrin ring from the original D_2h_ to D_4h_ [29,33].

### 2.4. X-Ray Photoelectron Spectroscopy

We performed X-ray photoelectron spectroscopy (XPS) to verify the oxidation states of Ni and Pd in **Pd_6_-TMPP(Ni)**. The XPS shows that in the Pd 3d region, peaks at binding energies of 337.5 ± 0.5 eV and 343.8 ± 0.6 eV were assigned to Pd 3d_5/2_ and Pd 3d_3/2_ (Figure 1c), which were in line with the characteristic peaks of Pd^2+^ [34]. Meanwhile, in the Ni 2p region, peaks at binding energies of 855.3 ± 0.8 eV and 872.5 ± 0.9 eV corresponded to the spin–orbit split peaks of Ni 2p_3/2_ and Ni 2p_1/2_ (Figure 1d), consistent with those reported in the literature [35]. The above data collectively confirmed that both Pd and Ni existed in the +2 oxidation state in **Pd_6_-TMPP(Ni)**.

### 2.5. Matrix-Assisted Laser Desorption/Ionization Time-of-Flight Mass Spectrometry

We used the matrix-assisted laser desorption/ionization time-of-flight mass spectrometry (MALDI-TOF MS) to confirm the molecular weight of **Pd_6_-TMPP(Ni)**. In the MALDI-TOF MS (Figure 1f and Figure S2), the peaks at a mass-to-charge ratio (*m*/*z*) of 3258.421 and 3280.443 were observed for **Pd_6_-TMPP(Ni)**, consistent with those calculated for [M+H]^+^ (3258.706 *m*/*z*) and [M + Na]^+^ (3280.695 *m*/*z*).

### 2.6. Energy-Dispersive X-Ray Spectroscopy

We used the energy-dispersive X-ray spectroscopy (EDS) to further validate the elemental composition of **Pd_6_-TMPP(Ni)**. As shown in Figure S3, the atomic ratio of Pd to Ni in **Pd_6_-TMPP(Ni)** was approximately 4.1:1.9 (equal to 2.2:1.0), which was close to the theoretical ratio in its molecular formula (2.0:1.0), suggesting the correct compositions of TMPP(Ni) and PdCl_2_ units and thus the whole cage.

### 2.7. Fourier-Transform Infrared Spectroscopic Characterization

**Figure 2:**
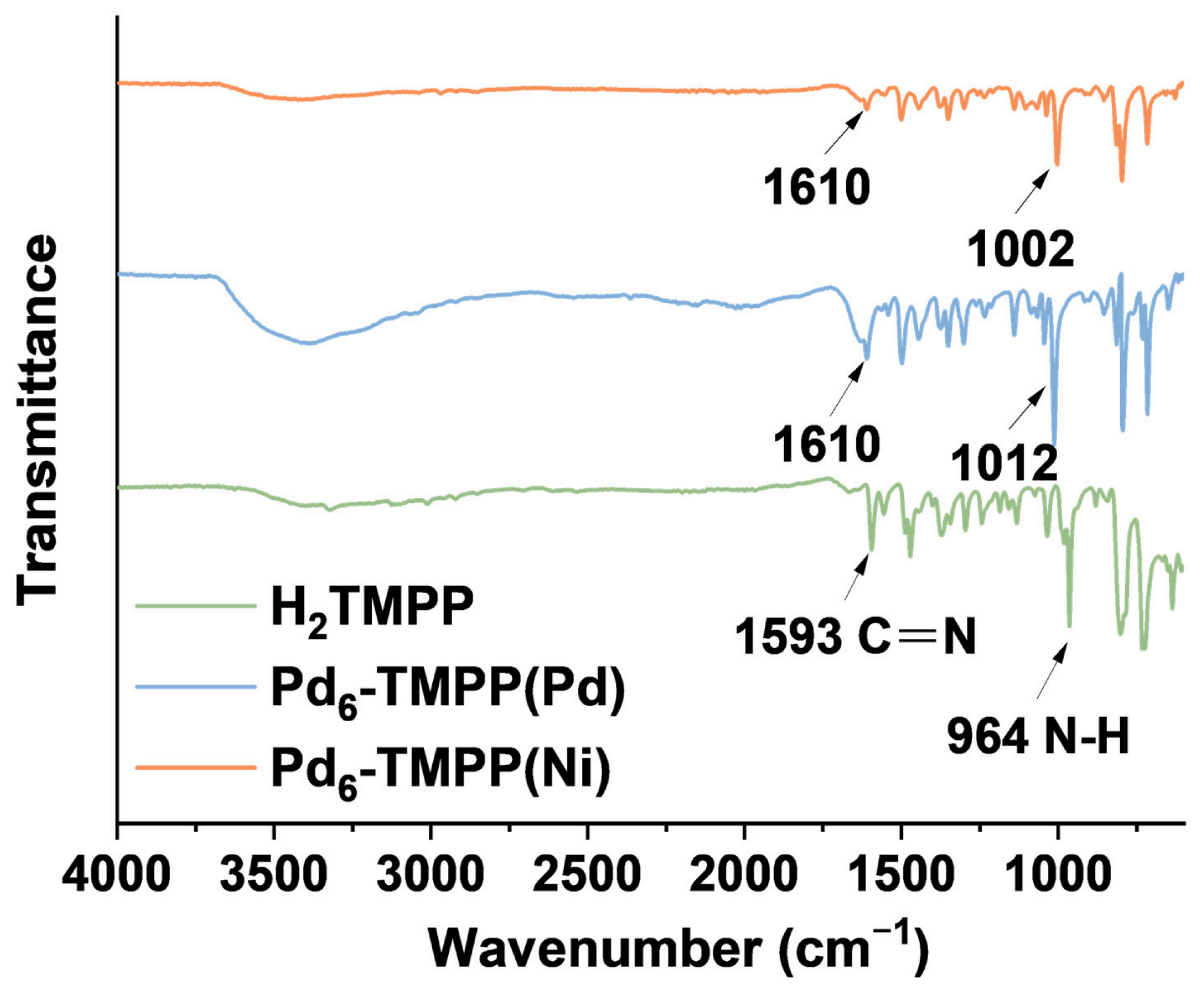
A comparison of the FT-IR spectra of **H_2_TMPP**, **Pd_6_-TMPP(Pd)**, and **Pd_6_-TMPP(Ni)**.

In order to study the variations in the characteristic vibration bands, we compared the Fourier-transform infrared (FT-IR) spectra of **H_2_TMPP**, **Pd_6_-TMPP(Pd)**, and **Pd_6_-TMPP(Ni)**. In the FT-IR spectra (Figure 2), the characteristic peak attributed to the in-plane vibration of N-H at the porphyrin center (964 cm^−1^) in **H_2_TMPP** disappeared in **Pd_6_-TMPP(Ni)**, while new peaks appeared at 1002 cm^−1^ and 1012 cm^−1^, which directly confirmed that Ni^2+^ had replaced the protons at the porphyrin center [36,37]. In addition, the stretching vibration peak of the C=N bond in the porphyrin ring was also red-shifted from 1593 cm^−1^ in **H_2_TMPP** to 1610 cm^−1^ in **Pd_6_-TMPP(Ni)** due to the coordination of pyridine nitrogen atoms with Pd^2+^ [38,39].

### 2.8. Synthesis and Characterizations of Pd_6_-TMPP(Ni) Nanoparticles

**Figure 3:**
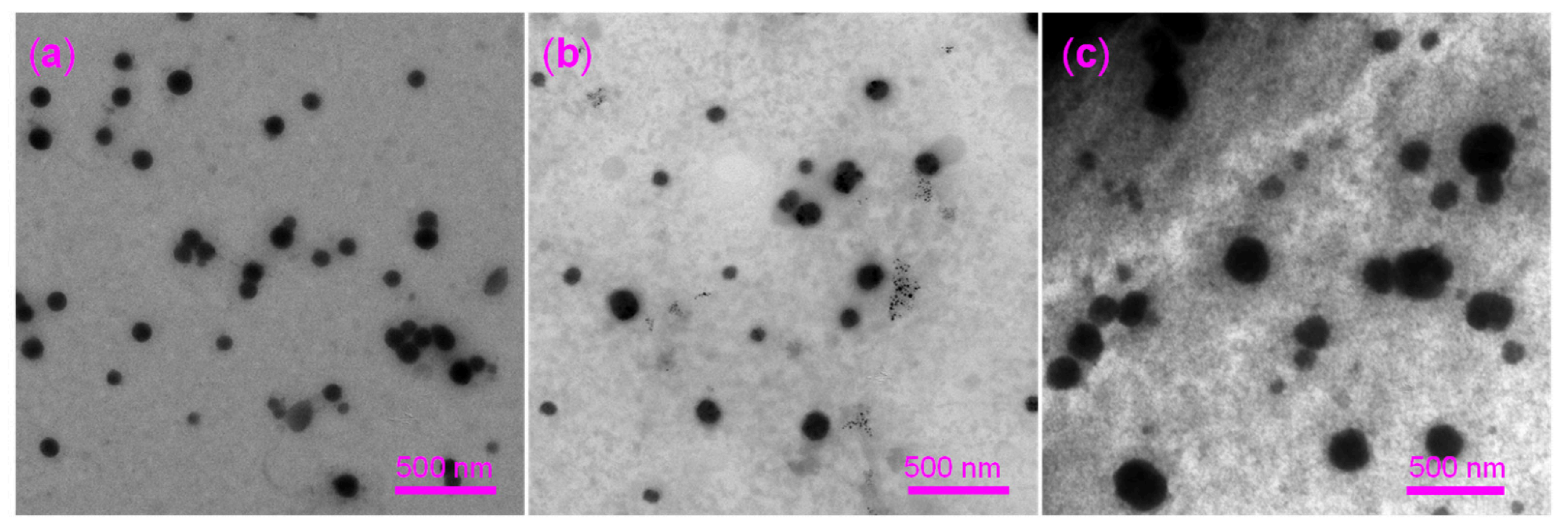
The TEM images of (**a**) **H_2_TMPP-F127**, (**b**) **Pd_6_-TMPP(Pd)-F127**, and (**c**) **Pd_6_-TMPP(Ni)-F127**.

Water-soluble nanoparticles were prepared by encapsulating **H_2_TMPP**, **Pd_6_-TMPP(Pd)**, and **Pd_6_-TMPP(Ni)** with Pluronic F127, an FDA-approved triblock polymer for therapeutic use (Scheme 1b; FDA denotes U.S. Food and Drug Administration) [40,41], in a mixture of DMSO and H_2_O, followed by dialysis against deionized H_2_O. The transmission electron microscopy (TEM) showed that the obtained nanoparticles **H_2_TMPP-F127**, **Pd_6_-TMPP(Pd)-F127**, and **Pd_6_-TMPP(Ni)-F127** all exhibited regular spherical morphology and good dispersibility in water (Figure 3), with their diameters being 190 ± 15 nm, 140 ± 15 nm, and 230 ± 15 nm, respectively. The hydrodynamic diameters as revealed by dynamic light scattering (DLS) were marginally larger due to the hydration of these nanoparticles, featuring 231.9 nm for **H_2_TMPP-F127**, 184.6 nm for **Pd_6_-TMPP(Pd)-F127**, and 311.7 nm for **Pd_6_-TMPP(Ni)-F127** (Figure S4). It is also notable that, as compared to the blank F127 (139.9 nm; Figure S4), the particle sizes are marginally enlarged, likely due to the encapsulation of additional molecular species.

Zeta potential was used to study the colloidal stability of these particles in aqueous solutions. The zeta potentials of these nanoparticles were 9.6 mV for **H_2_TMPP-F127**, 14.9 mV for **Pd_6_-TMPP(Pd)-F127**, and 27.4 mV for **Pd_6_-TMPP(Ni)-F127**, respectively (Figure S5). The highest zeta potential value of **Pd_6_-TMPP(Ni)-F127**, as compared to **H_2_TMPP-F127** and **Pd_6_-TMPP(Pd)-F127**, indicates its best colloidal stability, which is critical during the drug delivery stage [42,43].

### 2.9. Detection of •OH as Induced by H_2_TMPP-F127, Pd_6_-TMPP(Pd)-F127, and Pd_6_-TMPP(Ni)-F127 in Solution

**Figure 4:**
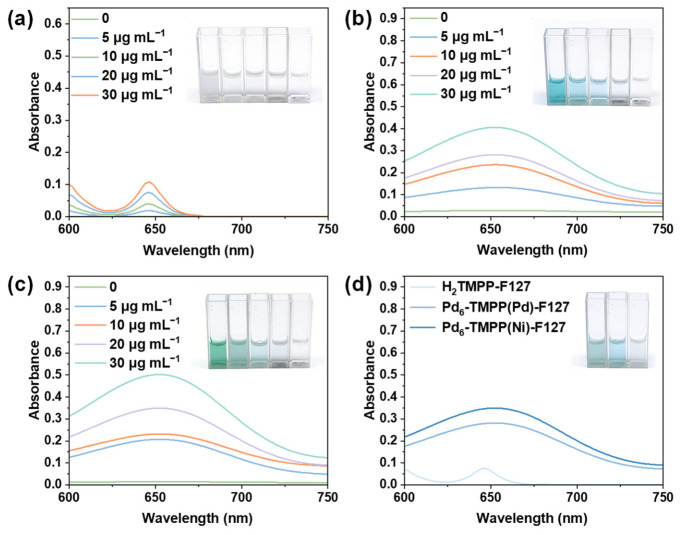
Characterization of •OH generation. The UV-Vis absorbance intensity at 652 nm (corresponding to oxTMB) after treatment with gradient TMPP concentrations of (**a**) **H_2_TMPP-F127**, (**b**) **Pd_6_-TMPP(Pd)-F127**, and (**c**) **Pd_6_-TMPP(Ni)-F127** in the presence of 100 μM H_2_O_2_, showcasing the catalytic generation of •OH. (**d**) The absorbance intensity at 652 nm upon treatment of fixed TMPP concentration (20 μg·mL^−1^) of **H_2_TMPP-F127** (light blue), **Pd_6_-TMPP(Pd)-F127** (blue), and **Pd_6_-TMPP(Ni)-F127** (dark blue) with 100 μM H_2_O_2_.

The generation of •OH can be chemically assayed by 3,3′,5,5′-tetramethylbenzidine (TMB) [22,44,45,46]. As shown in Figure 4, when **H_2_TMPP-F127**, **Pd_6_-TMPP(Pd)-F127**, and **Pd_6_-TMPP(Ni)-F127** were respectively mixed with a TMB solution containing 100 μM H_2_O_2_, **H_2_TMPP-F127** produced no •OH within 3 h, while the amount of •OH generated by **Pd_6_-TMPP(Pd)-F127** and **Pd_6_-TMPP(Ni)-F127** increased with the rise in concentration. At the same TMPP concentration, **Pd_6_-TMPP(Ni)-F127** produced significantly more •OH, which may be attributed to the fact that Ni(II) can activate the Fenton-like reaction to generate cytotoxic •OH [9].

**Figure 5:**
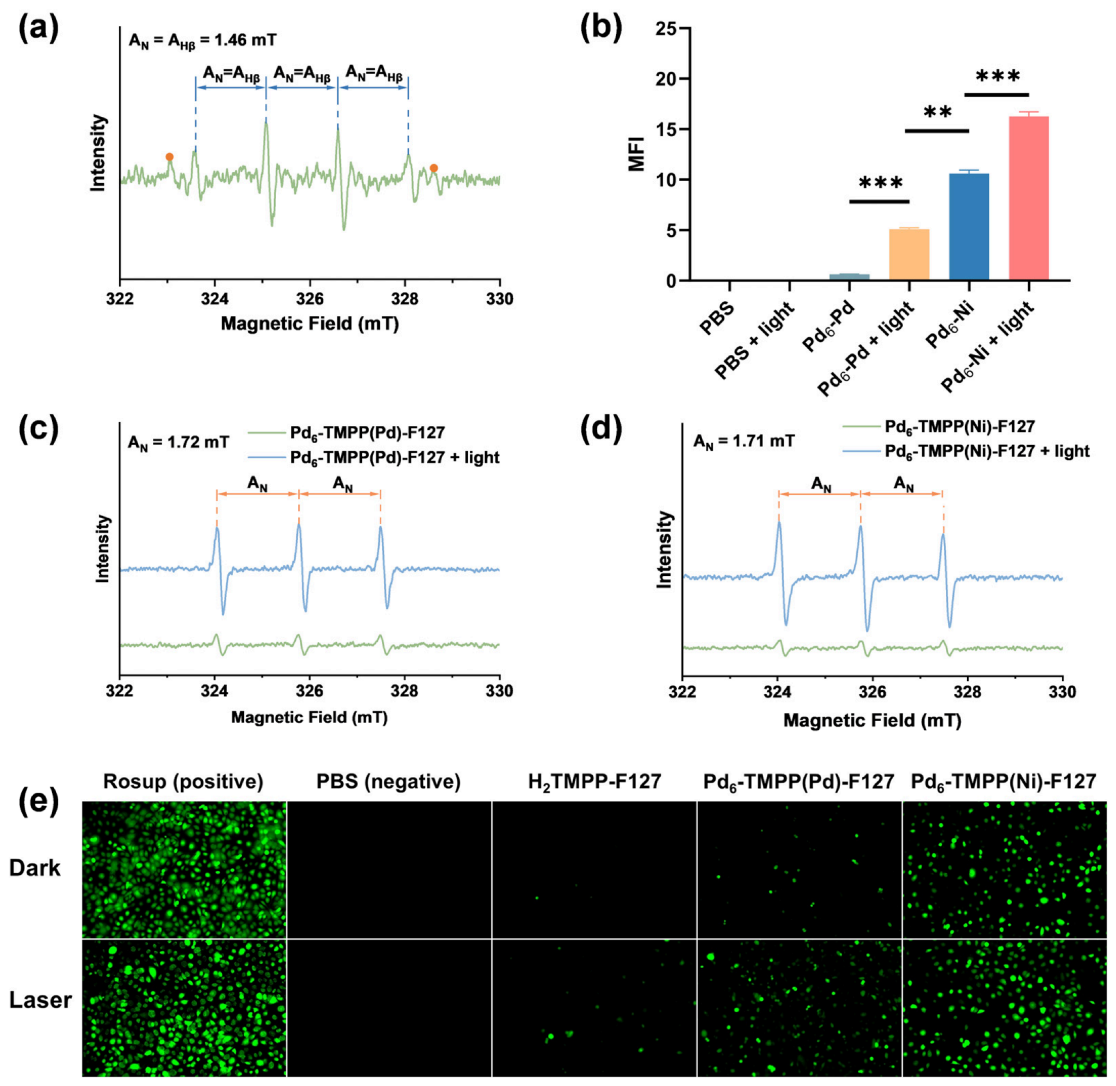
ROS generation. (**a**) The EPR spectra for •OH as induced by **Pd_6_-TMPP(Ni)-F127**, using DMPO as a spin trap (central magnetic field: 326 mT; tuning frequency: 10 GHz; the peaks marked with orange spots are ascribed to the methyl radicals from the TMPP ligand). (**b**) Comparison of the corresponding mean fluorescence intensity (MFI) of **Pd_6_-TMPP(Pd)-F127** (denoted as **Pd_6_-Pd**) and **Pd_6_-TMPP(Ni)-F127** (denoted as **Pd_6_-Ni**) after 24 h of culture in Te-1 cells with/without 650 nm light irradiation, using PBS as a negative control. The EPR spectra showing the ^1^O_2_ generation in the presence/absence of light irradiation as induced by (**c**) **Pd_6_-TMPP(Pd)-F127** and (**d**) **Pd_6_-TMPP(Ni)-F127**, using TEMP as the spin trap (central magnetic field: 326 mT; tuning frequency: 10 GHz). (**e**) Comparison of the intracellular ROS-generating ability of **H_2_TMPP-F127**, **Pd_6_-TMPP(Pd)-F127**, and **Pd_6_-TMPP(Ni)-F127** in Te-1, in the presence/absence of laser irradiation. Data are presented as mean ± s.d. ** *p* < 0.01, *** *p* < 0.001.

As shown in Figure 5a and Figure S6, the electron paramagnetic resonance (EPR) spectroscopy for both **Pd_6_-TMPP(Pd)-F127** and **Pd_6_-TMPP(Ni)-F127** using DMPO as a spin trap exhibited four peaks with a intensity ratio of 1:2:2:1 (DMPO = 5,5-dimethyl-1-pyrroline N-oxide), which confirmed the presence of •OH induced by both types of metal ions wherein Ni(II) outperforms Pd(II) [47,48,49]. It is however notable that, apart from the main signal for DMPO-OH (A_N_ = A_Hβ_ = 1.46 mT; total width is approximately 4.48 mT; g = 2.0055) [50], there also exist a pair of very weak peaks (Figure 5a, marked with orange spots) beyond these signals with a total width of around 5.53 mT, which is probably due to the formation of methyl radicals (from TMPP ligand) that are captured by DMPO [51]. Nevertheless, we are not able to isolate a complete peak set (sextets) due to the high overlap of the additional peaks with the strong DMPO-OH signals, apart from their low signal-to-noise ratio. The above experiments showed that both **Pd_6_-TMPP(Ni)-F127** and **Pd_6_-TMPP(Pd)-F127** could induce the chemodynamic therapeutic effect by generating •OH, whereas **Pd_6_-TMPP(Ni)-F127** was superior to **Pd_6_-TMPP(Pd)-F127** in this aspect.

### 2.10. Detection of ^1^O_2_ as Induced by H_2_TMPP-F127, Pd_6_-TMPP(Pd)-F127, and Pd_6_-TMPP(Ni)-F127 Under Light Irradiation

**Figure 6:**
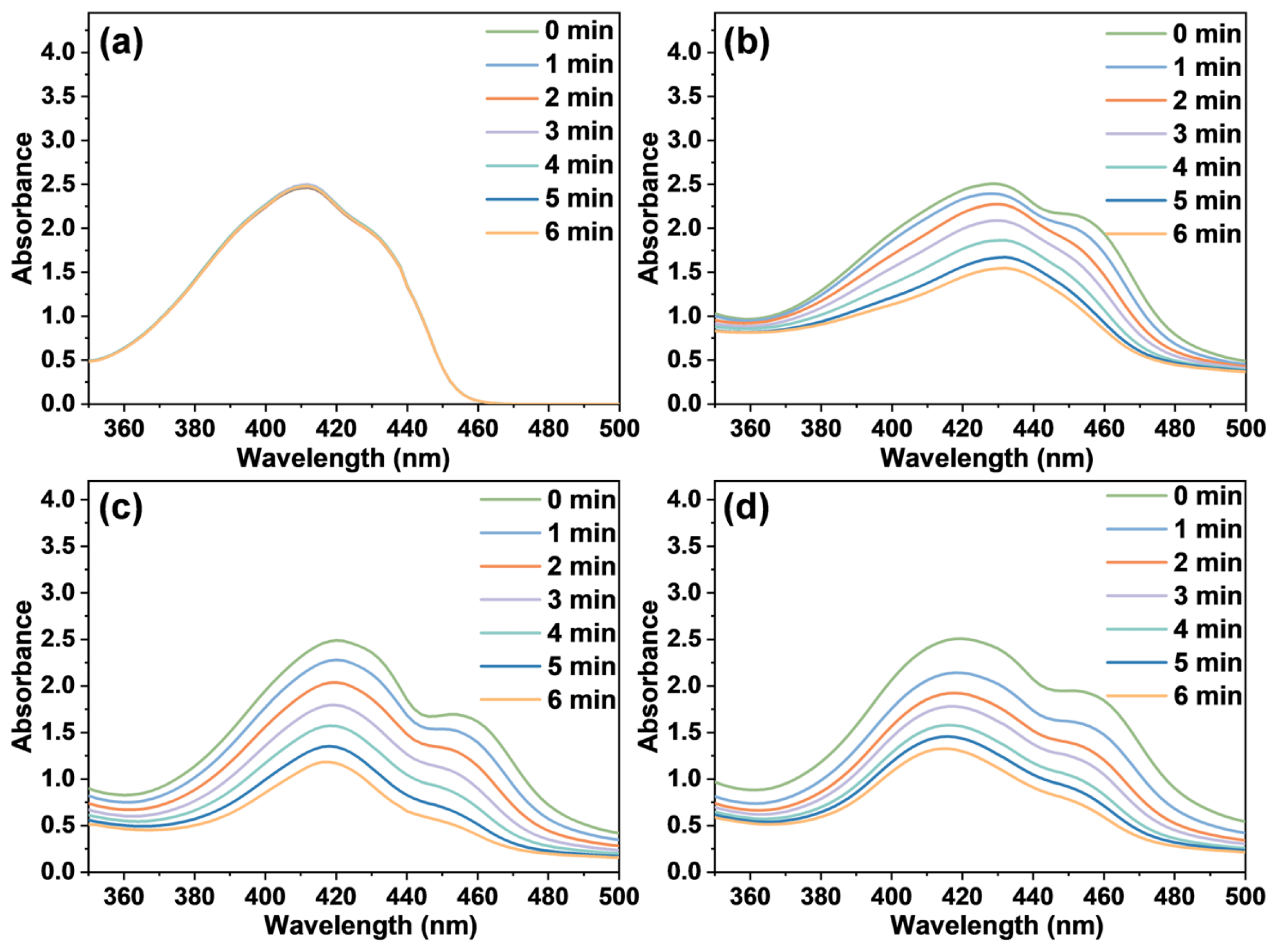
Photoinduced ^1^O_2_ generation. The intensity changes in the characteristic absorption band of DPBF at 416 nm in the presence of different photosensitizers: (**a**) free DPBF control, (**b**) **H_2_TMPP-F127**, (**c**) **Pd_6_-TMPP(Pd)-F127**, and (**d**) **Pd_6_-TMPP(Ni)-F127**.

We further used 1,3-diphenylisobenzofuran (DPBF) as a probe to detect and compare the ability of **H_2_TMPP-F127**, **Pd_6_-TMPP(Pd)-F127**, and **Pd_6_-TMPP(Ni)-F127** to generate ^1^O_2_ in aqueous solutions [52,53]. As shown in Figure 6a–d and Figure S7a, the absorbance of DPBF decreased after 1 min of irradiation, with the decrease rate being similar among these particles.

EPR measurements using 2,2,6,6-tetramethylpiperidine (TEMP) as the trapping agent showed that both **Pd_6_-TMPP(Ni)-F127** and **Pd_6_-TMPP(Pd)-F127** could induce the generation of ^1^O_2_ (Figure 5c,d), with their spectra exhibiting characteristic triplet featuring an intensity ratio of 1:1:1 (A_N_ = 1.71–1.72 mT; total width of around 3.42–3.44 mT; g = 2.0056) [54,55]. Notably, the peak intensity under light conditions was significantly higher than that in the dark for both materials, highlighting the effective ^1^O_2_ generation for PDT.

### 2.11. Particle Stability Characterizations

In order to obtain the stability profiles of the prepared particles. We investigated the stability of these particles under different experimental conditions. As shown in Figure S7b, the Soret bands of **H_2_TMPP-F127**, **Pd_6_-TMPP(Pd)-F127**, and **Pd_6_-TMPP(Ni)-F127** remained constant when exposed to a 650 nm laser for 10 min, suggesting that these particles are photostable. In addition, these particles were stable in H_2_O, PBS (0.1×), and RPMI 1640 (0.1×) for 0, 8, 12, and 24 h (PBS = phosphate-buffered saline; RPMI = Roswell Park Memorial Institute), indicated by only insignificant changes in particle size as revealed by DLS (Figure S8).

### 2.12. Cell Cytotoxicity Assay

**Figure 7:**
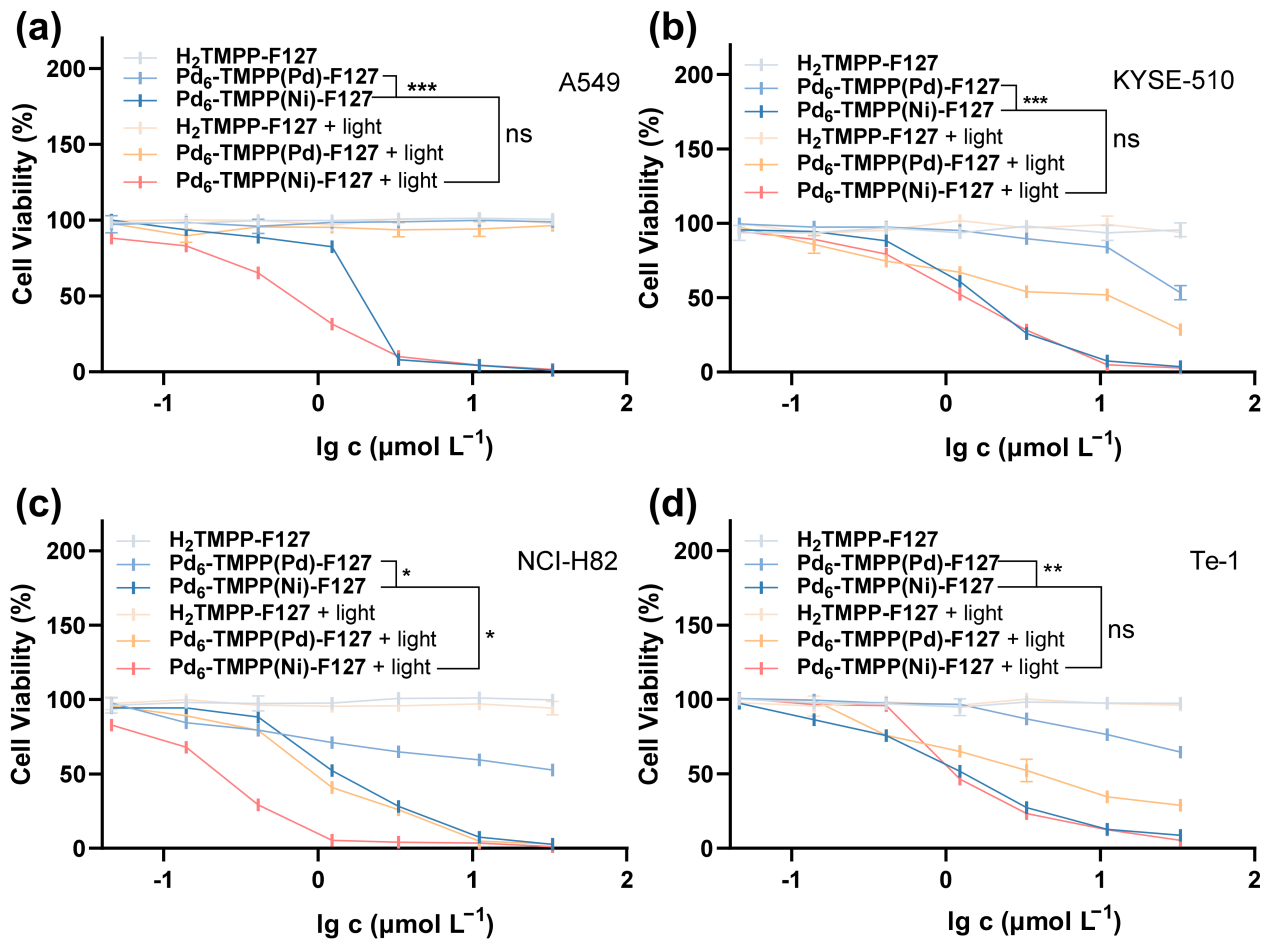
Cytotoxicity assay by CCK-8. Cell viability and data curves of (**a**) A549, (**b**) KYSE-510, (**c**) NCI-H82, and (**d**) Te-1 incubated with **H_2_TMPP-F127**, **Pd_6_-TMPP(Pd)-F127**, and **Pd_6_-TMPP(Ni)-F127** at gradient concentrations under conditions with/without laser irradiation (650 nm, 25 mW cm^−2^, 5 min). Data are presented as mean ± s.d. * *p* < 0.05, ** *p* < 0.01, *** *p* < 0.001; ns = not significant.

The cytotoxicity of **H_2_TMPP-F127**, **Pd_6_-TMPP(Pd)-F127**, and **Pd_6_-TMPP(Ni)-F127** against A549 (Figure 7a), KYSE-510 (Figure 7b), NCI-H82 (Figure 7c), and Te-1 (Figure 7d) cells under light and light-free conditions was evaluated and compared. For all these cell lines, **H_2_TMPP-F127** shows negligible cytotoxicity under both light and light-free conditions. In contrast, both **Pd_6_-TMPP(Pd)-F127** and **Pd_6_-TMPP(Ni)-F127** show concentration-dependent cytotoxicity against all these cell lines, with the exception that A549 is insensitive to **Pd_6_-TMPP(Pd)-F127** treatment within the tested concentration range. **Pd_6_-TMPP(Ni)-F127** also outperforms **Pd_6_-TMPP(Pd)-F127** for all these cell lines in terms of both dark and light toxicities, with **Pd_6_-TMPP(Ni)-F127** nearly killing all the cells with its TMPP concentrations above 3.33 μmol L^−1^ in the dark (equivalent to 1.11 μmol L^−1^ of **Pd_6_-TMPP(Ni)**). It is also feasible that light irradiation further augments the toxicity of both **Pd_6_-TMPP(Pd)-F127** and **Pd_6_-TMPP(Ni)-F127**, albeit for **Pd_6_-TMPP(Ni)-F127**, the light contribution to the toxicity against esophageal cancer cell lines KYSE-510 and Te-1 is insignificant. The low PDT contribution of porphyrin-chelated Ni(II) compared to Pd(II) can be explained by the fact that Ni^2+^ can quench the excited state of TMPP ligand via fast non-radiative decay due to the presence of metal-to-ligand charge transfer (MLCT) and d-d excited states that lie below or close to the π-π* states of TMPP [56,57].

The half maximal inhibitory concentration (IC_50_) for **Pd_6_-TMPP(Ni)-F127** against cell lines NCI-H82 (dark: 2.0 μmol L^−1^; light: 1.1 μmol L^−1^), A549 (dark: 2.0 μmol L^−1^; light: 0.8 μmol L^−1^), KYSE-510 (dark: 2.1 μmol L^−1^; light: 1.5 μmol L^−1^), and Te-1 (dark: 1.9 μmol L^−1^; light: 1.2 μmol L^−1^) are comparable to those reported in the literature, such as SnL_b_Cl_2_ (3.9 µM, MCF-7, light) [58], GaL_c_ (14.1 µM, Colon 26, light) [59], and MnL_f_(H_2_O)_2_ (6.2 µM, HeLa, light) [60] (Table 2), likely due to the presence of shared treatment modalities (i.e., ^1^O_2_ and •OH). It is also notable that the combination of CDT and PDT for **Pd_6_-TMPP(Ni)-F127** outperforms those materials solely based on PDT, such as ZnL_g_ (160.0 µM, A549, light) [61] and ZnL_h_ (184.5 µM, A549, light) [62].

### 2.13. Vitro ROS Generation Assay

To investigate the ROS generation capability of the particles in cells, 2′,7′-dichlorodihydrofluorescein diacetate (DCFH-DA) was used as a fluorescent probe [63]. DCFH-DA itself is non-fluorescent and can freely cross the cell membrane into cells. Once inside cells, it is hydrolysed by intracellular esterase to produce 2′,7′-dichlorodihydrofluorescein (DCFH), which can no longer pass through the cell membrane and thus becomes “locked” inside the cells. When ROS are present in the cells (H_2_O_2_, ^1^O_2_, O_2_^•−^, •OH, etc.), DCFH is oxidized to 2′,7′-dichlorofluorescein (DCF), which exhibits green fluorescence upon irradiation (λ_ex_/_em_ = 488/525 nm) [18,28,64]. As shown in Figure 5b,e, in the absence of laser irradiation, both **Pd_6_-TMPP(Pd)-F127** and **Pd_6_-TMPP(Ni)-F127** were able to generate a certain level of ROS. The presence of Ni in **Pd_6_-TMPP(Ni)-F127** further enhanced this activity. After laser irradiation, the amount of ROS generated by **Pd_6_-TMPP(Pd)-F127** and **Pd_6_-TMPP(Ni)-F127** increased significantly, while **H_2_TMPP-F127** produced almost no ROS.

### 2.14. Cellular Uptake of Pd_6_-TMPP(Pd)-F127 and Pd_6_-TMPP(Ni)-F127

We used inductively coupled plasma mass spectrometry (ICP-MS) to compare the cellular uptake efficacy of **Pd_6_-TMPP(Pd)-F127** and **Pd_6_-TMPP(Ni)-F127**. As shown in Table 3, both particles can be internalized by KYSE-510 cells in a time-dependent manner. It is also notable that the uptake efficiency of **Pd_6_-TMPP(Ni)-F127** was higher than that of **Pd_6_-TMPP(Pd)-F127**, which probably also contributed to the higher cytotoxicity of **Pd_6_-TMPP(Ni)-F127** observed in the cytotoxicity assays.

**Table 2:** IC_50_ values (μmol L^−1^) of **H_2_TMPP-F127**, **Pd_6_-TMPP(Pd)-F127**, and **Pd_6_-TMPP(Ni)-F127** against different cell lines in the presence/absence of laser irradiation.

Entry	Compound	Cell Line	Dark/Light	IC_50_ (µM)	Reference
1	[PdL_a_Cl]_2_	MCF-7	Dark	26.1	[65]
2	SnL_b_Cl_2_	MCF-7	Light	3.9	[58]
3	GaL_c_	Colon 26	Light	14.1	[59]
4	AuL_d_	A2780	Dark	0.2	[66]
5	ZnL_e_	HeLa	Dark	8.8	[67]
6	MnL_f_(H_2_O)_2_	HeLa	Light	6.2	[60]
7	ZnL_g_	A549	Light	160.0	[61]
8	ZnL_h_	A549	Light	184.6	[62]
9	RuL_j_L_k_Cl	A549	Light	12.7	[68]
10	Cu-TCPP(Fe)	A549	Dark	194.5	[69]
11	NiL_l_	HepG2	Dark	21.4	[70]
12	**Pd_6_-TMPP(Ni)-F127**	NCI-H82	Dark	2.0	This work
13	**Pd_6_-TMPP(Ni)-F127**	NCI-H82	Light	1.1	This work
14	**Pd_6_-TMPP(Ni)-F127**	A549	Dark	2.0	This work
15	**Pd_6_-TMPP(Ni)-F127**	A549	Light	0.8	This work
16	**Pd_6_-TMPP(Ni)-F127**	KYSE-510	Dark	2.1	This work
17	**Pd_6_-TMPP(Ni)-F127**	KYSE-510	Light	1.5	This work
18	**Pd_6_-TMPP(Ni)-F127**	Te-1	Dark	1.9	This work
19	**Pd_6_-TMPP(Ni)-F127**	Te-1	Light	1.2	This work

L_a_ = N-methyl-N’-(3-methylpyridin-2-yl)-1-(l1-sulfaneyl)methanediamine; L_b_ = 5,10,15,20-tetrakis(4-(methylthio)phenyl)porphyrin; L_c_ = 5,10,15,20-tetra(pyridin-4-yl)porphyrin; L_d_ = 5,10,15,20-tetraphenylporphyrin; L_e_ = N-((9,10-dioxo-9,10-dihydroanthracen-1-yl)carbamoyl)-2-(4-(10,15,20-tri-o-tolylporphyrin-5-yl)phenoxy)acetamide; L_f_ = 5,10,15,20-tetra-p-tolylporphyrin; L_g_ = 4-(10,15,20-tris(4-chlorophenyl)porphyrin-5-yl)phenyl 2-((4-oxo-2-phenyl-4H-chromen-6-yl)oxy)acetate; L_h_ = 4-(10,15,20-tris(4-chlorophenyl)porphyrin-5-yl)phenyl 2-(4-allyl-2-methoxyphenoxy)propanoate; L_j_ = (E)-5-methyl-2-((phenylimino)methyl)aniline; L_k_ = p-cymene, L_l_ = 3,5-dimethoxy-5′-phenyl-[1,1′:3′,1′’-terphenyl]-4-ol.

**Table 3:** The time-dependent Pd concentrations (ppb) in KYSE-510 cells (1 × 10^7^ cells) with the administration of **Pd_6_-TMPP(Pd)-F127** and **Pd_6_-TMPP(Ni)-F127** at the TMPP concentration of 10 μmol L^−1^.

	Pd_6_-TMPP(Pd)-F127	Pd_6_-TMPP(Ni)-F127
2 h	25.3 ± 2.0	45.0 ± 3.3
4 h	38.4 ± 4.6	76.4 ± 4.6
6 h	50.3 ± 5.7	96.4 ± 9.6

### 2.15. A General Discussion of the Main Work

A discrete metallocage with well-defined stoichiometry, namely, **Pd_6_-TMPP(Ni)**, was constructed through coordination-driven self-assembly via integrating Ni(II)-chelated porphyrin metalloligands with Fenton-like reactivity and Pd(II) nodes. Single-crystal X-ray diffraction analysis confirmed that the cage adopts a hexagonal prismatic geometry and possesses an inherent hydrophobic internal cavity. Such structural characteristics endow **Pd_6_-TMPP(Ni)** with dual functions. On the one hand, the introduced Ni(II) centers can convert H_2_O_2_ into highly toxic •OH via Fenton-like reactions, achieving oxygen-independent CDT. On the other hand, the porphyrin backbone retains promising photophysical properties and can efficiently produce ^1^O_2_ under 650 nm laser irradiation to realize PDT. The design integrating metal catalytic and photosensitive centers into one molecular skeleton effectively avoids component separation and proportional imbalance in traditional nanocarriers via weak intermolecular interactions.

**Pd_6_-TMPP(Ni)** exhibits efficient ROS generation capability, which benefits from the catalytic activity of Ni(II), and is further augmented by the photosensitizing ability of the TMPP ligand. In cellular assays, **Pd_6_-TMPP(Ni)** exerted remarkable light-triggered cytotoxicity against multiple tumor cell lines, including lung cancer and esophageal cancer cells. Notably, the coordination cage exhibits inherent dark cytotoxicity originating from its intrinsic CDT effect even without laser irradiation. These results fully verify the synergistic enhancement effect between CDT and PDT.

## 3. Conclusions

In this work, we synthesized a nonanuclear Pd-based coordination cage of **Pd_6_-TMPP(Ni)** that elicits its anticancer effect via dual ROS generation strategies, viz., catalytic •OH via the Ni^2+^ center and the photoinduced ^1^O_2_ generation via the TMPP ligand. These results validate that **Pd_6_-TMPP(Ni)** is a promising candidate for deep-seated and hypoxia-tolerant cancer therapy. In view of the diverse transition and main groups of metal ions (i.e., Mn^2+^, Fe^2+^/Fe^3+^, Co^3+^, Cu^2+^, Zn^2+^) that can be readily chelated within the porphyrin center, which may result in diverse ROS generation mechanisms [54], our future work will focus on exploring the synthetic accessibility of **Pd_6_-TMPP(M)** and their ROS generation for cancer therapy. In addition to the direct ROS generation by the cage, these coordination cages are expected to further encapsulate guest molecules (i.e., C_60_) to modulate their ROS generation patterns [71]. Finally, the supramolecular assemblies formed can be equipped with targeting functions via coordination with tumor-specific ligands for more effective cancer therapies.

## Figures and Tables

**Table 1:** Crystal data and structure refinement parameters for **Pd_6_-TMPP(Ni)**.

	Pd_6_-TMPP(Ni)
Formula	C_132_H_96_Cl_12_N_24_Ni_3_Pd_6_
Formula Weight	3258.25
Crystal System	monoclinic
Space Group	*P*2_1_/*c*
*a*/Å	23.923(2)
*b*/Å	52.525(4)
*c*/Å	15.4675(13)
*α*/°	90
*β*/°	98.748(3)
*γ*/°	90
*V*/Å^3^	19,209(3)
*Z*	4
ρ_calc_/(g cm^–3^)	1.127
*F*(000)	6480
Total Reflections	226,697
Unique Reflections	29,517
Observations Reflections	18,465
Parameters	1510
*R_int_*	0.2495
*^a^ R*_1_ (*I* ≥ 2σ (*I*))	0.1056
*^b^ wR*_2_ (*I* ≥ 2σ (*I*))	0.2724
*^c^* GOF	1.073

*^a^ R*_1_ = Σ||*F*_o_| − |*F*_c_||/Σ|*F*_o_|, *^b^ wR*_2_ = {Σ[*w*(*F*_o_^2^ − *F*_c_^2^)^2^]/Σ[*w*(*F*_o_^2^)^2^]}^1/2^, *^c^* GOF = {Σ[*w*(*F*_o_^2^ − *F*_c_^2^)^2^]/(*n* − *p*)}^1/2^, where *n* is the number of reflections and *p* is total number of parameters refined.

## Data Availability

Data are contained within the article and Supplementary Materials.

## Supplementary Materials

The following supporting information can be downloaded at https://www.mdpi.com/article/10.3390/molecules31111874/s1. Detailed experimental procedures [28,32,41,72,73,74,75]. Figure S1: The ^1^H NMR spectra (400 MHz, 298 K, CDCl_3_) of **H_2_TMPP** (a), TMPP(Ni) (b), and **Pd_6_-TMPP(Ni)** (c). Figure S2: The full MALDI-TOF-MS spectra of **Pd_6_-TMPP(Ni)** using DCTB + TFA-Na as the composite matrix. Figure S3: The quantitative elemental ratios of powdery **Pd_6_-TMPP(Ni)** as characterized by EDS and elemental mapping diagrams showing the average distribution of elements. Figure S4: Particle size distribution diagrams of **H_2_TMPP-F127** (a), **Pd_6_-TMPP(Pd)-F127** (b), **Pd_6_-TMPP(Ni)-F127** (c), and blank F127 (d) as the control. Figure S5: Zeta potential diagrams of **H_2_TMPP-F127** (a), **Pd_6_-TMPP(Pd)-F127 (b)**, and **Pd_6_-TMPP(Ni)-F127** (c). Figure S6: The EPR spectra for •OH as induced by **Pd_6_-TMPP(Pd)-F127** and **Pd_6_-TMPP(Ni)-F127**, using DMPO as a spin trap (central magnetic field: 326 mT; tuning frequency: 10 GHz). Figure S7: The UV-Vis variation curves showing the differences of ^1^O_2_ generation efficiency (a), and the intensity change of the Soret bands in **H_2_TMPP-F127**, **Pd_6_-TMPP(Pd)-F127**, and **Pd_6_-TMPP(Ni)-F127** under continuous light irradiation (650 nm, 25 mW cm^−2^) for 10 minutes, showcasing their high photostability (b). Figure S8: The DLS diagram of **H_2_TMPP** (a), **Pd_6_-TMPP(Pd)-F127** (b), and **Pd_6_-TMPP(Ni)-F127** (c) in H_2_O, PBS (0.1×), RPMI 1640 (0.1×) for 0, 8, 12, and 24 h. Table S1: Relevant metal∙∙∙metal separations (Å), selected bond distances (Å), and bond angles (°) for **Pd_6_-TMPP(Ni)**.
