# Supplementary material for: A Nonanuclear Pd-Based Coordination Cage Featuring Ni-Chelated Porphyrin Ligand for Cancer Therapy via Combination of Chemodynamic and Photodynamic Modalities
Source: Molecules. 2026 May 29;31(11):1874. doi: 10.3390/molecules31111874 (PMC13258547; doi:10.3390/molecules31111874)
Supplement: Supplementary file 1 [file molecules-31-01874-s001.zip › molecules-4296093-supplementary.pdf]

Supplementary Material for

A Nonanuclear Pd-based Coordination Cage Featuring Ni-chelated Porphyrin Ligand for Cancer Therapy Via Combination of Chemodynamic and Photodynamic Modalities

Meng-Lin Dong [1], Ye Ning [1], Yuan-Hui Jia [1], Wen-Hua Zhang [1,*], Wenqiang Lu [2,*] and Yiming Mao [2,*]

[1] College of Chemistry, Chemical Engineering and Materials Science, Soochow University, Suzhou 215123, China

[2] Department of Thoracic Surgery, Suzhou Kowloon Hospital, Shanghai Jiao Tong University School of Medicine, Suzhou 215021, China

* Correspondence: whzhang@suda.edu.cn (W.H.Z.); 15050381628@163.com (W.L.); mym19850126@163.com (Y. M.)

Table of Contents

1. Materials and Methods 4

1.1. General 4

1.2. Synthesis of TMPP(Ni) 5

*1.3. Synthesis of **Pd_6-TMPP(Ni)*** 6

*1.4 Synthesis of **Pd_6-TMPP(Pd)*** 6

1.5. Single-crystal X-ray Crystallography 7

*1.6. The Formation of **H_2TMPP-F127**, **Pd_6-TMPP(Pd)-F127**, and **Pd_6-TMPP(Ni)-F127** Nanoparticles* 7

1.7 Photostability Assay 8

1.8 Hydroxyl Radical (•OH) Detection 8

1.9 Singlet Oxygen ($^1O_2$) Detection 8

1.10 CCK-8 Cytotoxicity Assay 9

1.11 Intracellular Reactive Oxygen Species Detection 10

1.12. Cellular Uptake 11

2. Supplementary Figures 12

Figure S1 The ^{1}H NMR spectra (400 MHz, 298 K, $CDCl_3$) of **H_2TMPP** (a), TMPP(Ni) (b), and **Pd_6-TMPP(Ni)** (c). 12

Figure S2 The full MALDI-TOF-MS spectra of **Pd_6-TMPP(Ni)** using DCTB + TFA-Na as the composite matrix. 13

Figure S3 The quantitative elemental ratios of powdery **Pd_6-TMPP(Ni)** as characterized by EDS and elemental mapping diagrams showing the average distribution of elements. 14

Figure S4 Particle size distribution diagrams of **H_2TMPP-F127** (a), **Pd_6-TMPP(Pd)-F127** (b), **Pd_6-TMPP(Ni)-F127** (c), and blank F127 (d) as the control. 15

Figure S5 Zeta potential diagrams of **H_2TMPP-F127** (a), **Pd_6-TMPP(Pd)-F127 (b)**, and **Pd_6-TMPP(Ni)-F127** (c). 16

Figure S6 The EPR spectra for •OH as induced by **Pd_6-TMPP(Pd)-F127** and **Pd_6-TMPP(Ni)-F127**, using DMPO as a spin trap (central magnetic field: 326 mT; tuning frequency: 10 GHz). 17

Figure S7 The UV-Vis variation curves showing the differences of $^1O_2$ generation efficiency

(a), and the intensity change of the Soret bands in **H_2TMPP-F127**, **Pd_6-TMPP(Pd)-F127**, and **Pd_6-TMPP(Ni)-F127** under continuous light irradiation (650 nm, 25 mW cm^{-2}) for 10 minutes, showcasing their high photostability (b). 18

Figure S8 The DLS diagram of **H_2TMPP** (a), **Pd_6-TMPP(Pd)-F127** (b), and **Pd_6-TMPP(Ni)-F127** (c) in H_2O, PBS (0.1×), RPMI 1640 (0.1×) for 0, 8, 12, and 24 h. 19

2. Supplementary Tables 20

Table S1 Relevant metal···metal separations (Å), selected bond distances (Å), and bond angles (°) for **Pd_6-TMPP(Ni)**. 20

3. References 22

1. Materials and Methods

1.1. General

Ligand **H_2TMPP** [1,2] was synthesized as described in our previous report. $PdCl_2$ (99%, TCI (Shanghai) Development Co., Ltd., Shanghai, China), $Ni(NO_3)_2·6H_2O$ (≥ 98%, Shanghai Aladdin Biochemical Technology Co., Ltd., Shanghai, China), Pluronic F-127 (MW: 2000, Shanghai Macklin Biochemical Co., Ltd, Shanghai, China), 3,3′,5,5′-tetramethylbenzidine (TMB, AR, Shanghai Yuanye Bio-Technology Co., Ltd., Shanghai, China), 1,3-diphenylisobenzofuran (DPBF, AR, Shanghai Maokang Biotechnology Co., Ltd., Shanghai, China), 5,5-dimethyl-1-pyrroline N-oxide (DMPO, 97%, Shanghai Aladdin Biochemical Technology Co., Ltd., Shanghai, China), 2,2,6,6-tetramethylpiperidine (TEMP, 95%, Shanghai Aladdin Biochemical Technology Co., Ltd., Shanghai, China), and hydrogen peroxide (30%, ≥ 95%, Yonghua Chemical Co., Ltd., Shanghai, China) were available from the corresponding suppliers without further purification. MeOH, MeCN, Et_2O, acetic acid (HOAc), dimethyl sulfoxide (DMSO), and N, N-dimethylformamide (DMF), all in analytical reagent (AR) grade, were procured from respective sources and used as received.

NCI-H82, A549, KYSE-510, and Te-1 cell lines were purchased from the Shanghai Institute of Cell Biology, Chinese Academy of Sciences (Shanghai, China). Phosphate-buffered solution (PBS), cell culturing medium RPMI 1640 (10% FBS + 1% P/S) and its FBS-free counterpart, Ham's F-12 (10% FBS + 1% P/S) and its FBS-free counterpart, and 0.25% trypsin solution in PBS (containing EDTA) were available from Shanghai Basal Media Technologies Co., Ltd (Shanghai, China). The ROS detection kit and DCFH-DA were available from Shanghai Beyotime Biotechnology Co., Ltd (Shanghai, China). The cell counting kit-8 (CCK-8) was available from APEXBIO.

The Fourier-transform infrared (FT-IR) spectra were collected from a Bruker Vertex 70 FTIR-spectrometer equipped with a Hyperion 2000 IR-Microscope (Bruker AXS GmbH, Germany), employing the ATR (attenuated total reflection) technique. The ^{1}H nuclear magnetic resonance (NMR) spectra were collected from a Bruker Avance III HD 400 MHz superconducting NMR spectrometer (Bruker AXS GmbH, Germany). Ultraviolet–visible (UV-Vis) spectra were acquired using a Varian Cary-50 UV-Vis spectrophotometer (Varian, Inc., Palo Alto, CA, USA). X-ray photoelectron spectroscopy (XPS) was performed on a

Thermo Scientific EXCALAB 250 XI X-ray photoelectron spectrometer (Thermo Scientific, Waltham, MA, USA). Energy-dispersive X-ray spectroscopy (EDS) was conducted using a Zeiss EVO 18 scanning electron microscope (ZEISS Group, Oberkochen, Germany). Transmission electron microscopy (TEM) images were obtained using a Hitachi HT7700 transmission electron microscope (Hitachi, Japan), and samples were prepared by dropping aqueous solutions onto copper grids. Dynamic light scattering (DLS) and zeta potential measurements were performed using a Horiba LA-95052 laser particle size analyzer (Horiba, Kyoto, Japan). The MALDI-TOF MS (matrix-assisted laser desorption/ionization time-of-flight mass) spectrometry was collected on a Bruker UltrafleXtreme MALDI-TOF/TOF (Bruker AXS GmbH, Germany) using DCTB + TFA-Na as the composite matrix (DCTB = *trans*-2-[3-(4-tert-butylphenyl)-2-methylprop-2-enyl]malononitrile; TFA-Na = sodium trifluoroacetate). Electron paramagnetic resonance (EPR) spectroscopy was carried out on a JEOL JES-X320 electron spin resonance spectrometer (JEOL Ltd., Akishima, Japan). Inductively coupled plasma–mass spectrometry (ICP-MS) was performed with an iCAP PRO instrument (Thermo Scientific, Waltham, MA, USA). Photodynamic therapy experiments were conducted using a PR-CPC2-635NM cellular phototoxicity irradiator at 650 nm (PURI Materials, Guangdong, China). Cytotoxicity measurements were conducted on a Tecan M1000PRO microplate reader (Tecan, Switzerland) by measuring the optical density at 450 nm. Fluorescence imaging of reactive oxygen species was conducted using an MF52 inverted biological microscope (Guangzhou Micro-shot Technology Co., Ltd., Guangdong, China).

1.2. Synthesis of TMPP(Ni)

$Ni(NO_3)_2{\cdot}6H_2O$ (2.0 mg, 6.88 μmol) and **H_2TMPP** (2.0 mg, 2.96 μmol) were introduced to a mixture of DMF/EtOH (v : v = 0.75 : 0.75 mL), and 2-pyridinemethanol (30 μL) was sequentially introduced as a modulator. The formed mixture was then added to a Pyrex glass tube (outer diameter 10 mm; inner diameter 6 mm; length 15 cm; same for the following reactions), sealed, and heated in a programmable oven, with the temperature smoothly increased from r.t. to 120°C in 4 hours. This is followed by a stable heating at 120°C for 36 hours before cooling back to r.t. within 12 hours to yield purple plate crystals of TMPP(Ni). The tubes (50 parallel reactions) were carefully opened, and the resulting product was

washed thoroughly with anhydrous ether and dried in an oven. Yield: 43.2 mg from 50 parallel reactions (39.8% based on Ni). FT-IR (ATR, cm^{-1}): 3050 (m), 3011 (w), 2922 (w), 2852 (w), 2162 (w), 1667 (w), 1595 (m), 1557 (w), 1487 (w), 1366 (m), 1349 (m), 1295 (m), 1249 (w), 1204 (w), 1132 (m), 1081 (w), 1036 (w), 1020 (w), 1000 (s), 884 (w), 863 (w), 798 (s), 732 (w), 716 (s), 649 (w), 612 (w). ^1H NMR (400 MHz, $CDCl_3$, ppm): δ 9.07 (d, *J* = 4.0 Hz, 4H), 8.69 (s, 8H), 8.13 (q, *J* = 8.0 Hz, 4H), 7.47 (d, *J* = 8.0 Hz, 4H), 2.81 (s, 12H).

1.3. Synthesis of ***Pd_6-TMPP(Ni)***

TMPP(Ni) (1.5 mg, 2.10 μmol) and $PdCl_2$ (1.0 mg, 5.63 μmol) were dissolved in a mixed solvent of CH_2Cl_2/MeOH (v : v = 0.5 : 1.5 mL). Acetic acid (HOAc, 20 μL) was subsequently introduced as a modulator. The resulting solution was transferred into a Pyrex glass tube, sonicated for 30 min, and sealed. The tube was then transferred to a programmable oven, with its temperature gradually increasing from r.t. to 120°C in 4 hours. This is followed by stable heating at 120°C for 48 hours, before cooling back to r.t. within 24 hours to yield red needle-like crystals of **Pd_6-TMPP(Ni)**. The tubes (70 parallel reactions) were carefully opened, and the resulting crystals were filtered off and washed with diethyl ether 2–3 times to afford **Pd_6-TMPP(Ni)**. Yield: 25.6 mg from 70 parallel reactions (16.4% based on **TMPP(Ni)**). FT-IR (ATR, cm^{-1}): 3323 (w), 3010 (w), 2923 (w), 1593 (m), 1548 (m), 1490 (s), 1471 (m), 1438 (w), 1384 (m), 1379 (m), 1338 (m), 1294 (m), 1240 (m), 1178 (m), 1151 (m), 1130 (m), 1085 (w), 1031 (m), 985 (m), 964 (s), 885 (m), 850 (m), 798 (s), 723 (s), 643 (s), 634 (m). UV-Vis (DMSO): λ_{max} (log ε) 417 nm (4.81), 525 nm (3.65), 559 nm (3.11). MALDI TOF-MS: m/z calcd for $[M+H]^+$: 3258.706, found 3258.421; calcd for $[M + Na]^+$: 3280.725, found 3280.443. ^1H NMR (400 MHz, $CDCl_3$, ppm): δ 9.36 (s, 12H), 8.72 (s, 12H), 8.54 (s, 12H), 8.26 (d, *J* = 8.0 Hz, 12H), 7.59 (d, *J* = 8.0 Hz, 12H), 3.76 (s, 36H).

1.4 Synthesis of ***Pd_6-TMPP(Pd)***

Pd_6-TMPP(Pd)-F127 was synthesized following our previous report [3]. Specifically, H_2TMPP (2 mg, 0.003 mmol) and $Pd(OAc)_2$ (2 mg, 0.009 mmol) in a CH_2Cl_2/MeOH mixed solvent (v : v = 0.5 mL : 1.5 mL) were transferred into a Pyrex glass tube. Subsequently, 20 μL of HOAc was introduced as a modulator was introduced. The glass tube was sealed and put

into a programmable oven, with its temperature increasing from 25°C to 120°C over 4 h. The temperature was maintained for 48 h before smoothly cooling down to 25°C within 24 h. The obtained solid from parallel reactions was re-dissolved in CH_2Cl_2 and filtered. The filtrate was concentrated by rotary evaporation, and MeOH was added to yield the precipitates, which were filtered off and washed with MeOH 2–3 times to afford **Pd_6-TMPP(Pd)**. IR (KBr disc; cm^{-1}): 3427 (m), 2921 (w), 1610 (m), 1546 (w), 1494 (s), 1447 (m), 1348 (m), 1296 (m), 1232 (w), 1132 (m), 1086 (m), 1045 (s), 1010 (vs), 911 (w), 853 (w), 818 (s), 789 (vs).

1.5. Single-crystal X-ray Crystallography

The single-crystal X-ray data for **Pd_6-TMPP(Ni)** were collected on a Bruker APEX II CCD (Bruker AXS GmbH, Germany) X-ray diffractometer equipped with Ga-Kα (λ = 1.34139 Å) irradiation. The data reduction and refinement were carried out on the program SAINT, with multi-scan applied for absorption corrections [4]. The structure of **Pd_6-TMPP(Ni)** was solved and refined with SHELXTL-2016 [5].

During refinement, some spatially delocalized electron density was observed within the crystal lattice, but it proved difficult to obtain satisfactory results. The solvent contribution was then removed using SQUEEZE in the Platon program suite [6].

The crystallographic data for **Pd_6-TMPP(Ni)** were submitted to CCDC (Cambridge Crystallographic Data Center) with the publication number 2525223. These data can be obtained via www.ccdc.cam.ac.uk/data_request/cif for free or directly as the Supplementary Material.

*1.6. The Formation of **H_2TMPP-F127**, **Pd_6-TMPP(Pd)-F127**, and **Pd_6-TMPP(Ni)-F127** Nanoparticles*

The formation of **H_2TMPP-F127** follows our previously published procedures [7]. Specifically, Pluronic F-127 (10.6 mg, 5.30 μmol) was introduced to a 1 mL DMSO containing **H_2TMPP** (5.00 mg, 7.41 μmol). Upon dissolution, the mixture was added dropwise into deionized water (10 mL) under tip sonication. The formed mixture was then dialyzed against deionized water (3 × 1000 mL) using a membrane with a molecular weight cutoff of 2000 Da for 24 h to obtain **H_2TMPP-F127** aqueous solution for subsequent use.

The preparation procedures for **Pd_6-TMPP(Pd)-F127** and **Pd_6-TMPP(Ni)-F127** were similar to that of **H_2TMPP-F127**, except that **Pd_6-TMPP(Pd)** (3.5 mg, 1.03 μmol) and **Pd_6-TMPP(Ni)** (3.5 mg, 1.07 μmol) were used as the starting material.

1.7 Photostability Assay

H_2TMPP-F127, **Pd_6-TMPP(Pd)-F127**, and **Pd_6-TMPP(Ni)-F127** aqueous solutions with the same porphyrin concentration (7 μg mL^{-1}) were prepared and then irradiated with 650 nm laser (25 mW cm^{-2}). The absorbance intensity at 416 nm was monitored over a period of 10 min, with data recorded every 1 min.

1.8 Hydroxyl Radical (•OH) Detection

Chemical method: Different concentrations of **H_2TMPP-F127**, **Pd_6-TMPP(Pd)-F127**, and **Pd_6-TMPP(Ni)-F127** were added to TMB solution containing 100 μM H_2O_2. After reacting for 3 hours at r.t., the reaction solutions were centrifuged to remove the influence of nanoparticles on absorbance. The UV-Vis curve of the supernatant at the wavelength of 652 nm was determined.

Spectroscopic method: **Pd_6-TMPP(Pd)-F127** and **Pd_6-TMPP(Ni)-F127** with the equivalent concentration of TMPP (7 μg mL^{-1}) were co-incubated with 100 μM H_2O_2, and after 3 hours, the spin trap 0.1 mL DMPO (97%) was added and stirred for 15 min. The resulting solution was then sealed in capillaries for EPR measurement.

1.9 Singlet Oxygen ($^1O_2$) Detection

The same concentration of DPBF (33 μg mL^{-1}) was added to aqueous solutions of **H_2TMPP-F127**, **Pd_6-TMPP(Pd)-F127**, and **Pd_6-TMPP(Ni)-F127** with an equivalent concentration of TMPP (7 μg mL^{-1}) to give uniform mixtures. The solutions were then irradiated with a 650 nm laser at an intensity of 25 mW cm^{-2}. After irradiation for every 1 min, the characteristic absorption intensity of DPBF at 416 nm was immediately measured.

Spectroscopic method: Equal concentrations of **Pd_6-TMPP(Pd)-F127** and **Pd_6-TMPP(Ni)-F127** (both with a TMPP concentration of 7 μg mL^{-1}) were used. For the phototoxicity experimental group, the system was first irradiated with a 650 nm laser (25 mW cm^{-2}) for 2

minutes, then 0.05 mL of the spin trap 2,2,6,6-tetramethylpiperidine (TEMP, 95%) was added and stirred for 10 minutes. For the dark toxicity control group, an equal amount of the spin trap (0.05 mL TEMP, 95%) was added directly and stirred for 1 minute. The resulting solutions were then sealed in capillaries for EPR measurement.

1.10 CCK-8 Cytotoxicity Assay

The suspension-grown cell line NCI-H82 was developed in RPMI 1640 medium (supplemented with 10% FBS + 1% P/S), or in its serum-free counterpart. To be specific, the cells were centrifuged, and the supernatant was discarded. The cells were then re-suspended in serum-supplemented RPMI 1640 at a concentration of 1×10^5 cells per milliliter, cultured at 37°C under a 5% CO_2 atmosphere.

For the CCK-8 assay, cells (3.5×10^4) were seeded in 100 μL of serum-free medium featuring gradient concentrations of **H_2TMPP-F127**, **Pd_6-TMPP(Pd)-F127**, and **Pd_6-TMPP(Ni)-F127**. Experiments were conducted with five replicates (n = 5) in a 96-well plate, using untreated cells as the 100% cell viability and culturing medium (RPMI 1640 + 10 μL PBS + 1% P/S + CCK-8) as the blank control.

For the PDT group, cells were gently centrifuged and rinsed with PBS three times, replenished with an equal volume of drug solutions, and then treated with a laser (650 nm, 25 mW cm^{-2}, 5 min) 24 h-post incubation, followed by an additional incubation time of 48 h. Meanwhile, the non-PDT group was incubated for 72 hours. After incubation, FBS (10 μL) and CCK-8 (10 μL) were introduced, and the cells were further incubated for 3.5 hours before analysis at 450 nm with a microplate reader. The relative cell viability (V%) was inferred from Equation (1) as shown below:

$$V\% = \frac{[A]_{experimental} - [A]_{blank}}{[A]_{control} - [A]_{blank}} \times 100\% \text{ (Equation 1)}$$

wherein V% denotes the percentage of cell viability, $[A]_{experimental}$ denotes the absorbance of the drug-treated cells, $[A]_{blank}$ denotes the absorbance of the blank control, and $[A]_{control}$ denotes the absorbance of the wells culturing untreated cells.

The adherent cells A549 were cultured in Ham's F-12 medium (supplemented with 10% FBS + 1% P/S), or its serum-free counterpart. Specifically, the cells were developed as a monolayer and then detached using trypsin (0.5% w/v in PBS). Upon trypsinization, the cells

were incubated for 3 min and centrifuged. A 3 mL of serum-supplemented culture medium was subsequently introduced to neutralize any residual trypsin. Cells were re-suspended in serum-supplemented medium at a concentration of 1×10^5 cells per milliliter and cultured (37°C, 5% CO_2) for the CCK-8 assay.

Cells (1×10^4) were seeded in 100 μL culture medium in a 96-well plate and incubated for 24 hours (37°C and 5% CO_2) for attachment. The culture medium was then replenished with serum-free medium containing gradient concentrations of **H_2TMPP-F127**, **Pd_6-TMPP(Pd)-F127**, and **Pd_6-TMPP(Ni)-F127**. All experiments were performed with five replicates (n = 5), using a protocol similar to that for NCI-H82.

For the PDT experiment, cells were rinsed with PBS three times and then replenished with an equal volume of drug solutions, and then treated with a laser (650 nm, 25 mW cm^{-2}, 5 min) after 24 h of incubation, followed by an additional incubation of 48 h. For the non-PDT group, cells were directly incubated for 72 h. Upon incubation, 100 μL of culture medium and 10 μL of CCK-8 were added, and the plates were incubated for an additional 1 h before analysis at 450 nm using a microplate reader. The relative cell viability (V%) of each group was calculated using Equation 1.

The cytotoxicity assessment for adherent cell lines KYSE-510 and Te-1 follows a similar protocol to that of the A549 cells, except that the culture media are different. Specifically, KYSE-510 cells were cultured in a 1 : 1 (v : v) mixture of RPMI 1640 and Ham's F-12 media, while Te-1 cells were cultured directly in RPMI 1640 medium.

1.11 Intracellular Reactive Oxygen Species Detection

Te-1 cells (8×10^5) were seeded in a 6-well plate with 2 mL serum-supplemented culture medium (RPMI 1640: Ham's F-12 = 1: 1 + 10% FBS + 1% P/S). The cells were incubated (37°C under 5% CO_2) for 24 h to allow attachment. Upon incubation, the culture medium was removed and then replenished with 1.5 mL serum-free medium (RPMI 1640 : Ham's F-12 = 1 : 1 + 1% P/S) containing **H_2TMPP-F127**, **Pd_6-TMPP(Pd)-F127**, and **Pd_6-TMPP(Ni)-F127** at a concentration of 10 μmol L^{-1} for each drug.

For the PDT group, cells were irradiated (650 nm, 25 mW cm^{-2}, 5 min), while cells in the non-PDT group were not irradiated. Cells in the PDT group were further incubated for 24 h

after irradiation, followed by the addition of 2 mL of fresh medium to each well. For the negative control wells, 1.0 μL of PBS was added, while for the negative control wells, 1.0 μL of Rosup-containing medium was added. After incubation for 30 min, 1 μL of DCFH-DA was added to each well, and the culture was continued for another 20 min. The cells were then rinsed three times with serum-free medium and then subjected to fluorescent performance measurement using an inverted fluorescence microscope.

1.12 Cellular Uptake

KYSE-510 cells (1×10^7) were seeded in cell culture dishes (9 parallel dishes) with a volume of 10 mL culture medium. After the cells adhered to the dish and developed to 95% confluency, the medium was replenished with serum-free medium. **Pd_6-TMPP(Pd)-F127** and **Pd_6-TMPP(Ni)-F127** were then introduced directly to the medium with a porphyrin concentration of 10 μmol L^{-1}. Upon drug administration, the cells were incubated for 2 h, 4 h, and 6 h, with three replicate groups (n = 3).

After incubation, cells were rinsed three times with PBS, confluence by trypsin digestion, transferred to 15 mL centrifuge tubes, and then centrifuged at 900 rpm for 3 min. The collected cells were rinsed twice with PBS buffer and centrifuged. The cells were then lysed with 1 mL of concentrated HNO_3 and then diluted to 10 mL with deionized H_2O and filtered. The intracellular contents of Pd and Ni were analyzed by ICP-MS.

2. Supplementary Figures

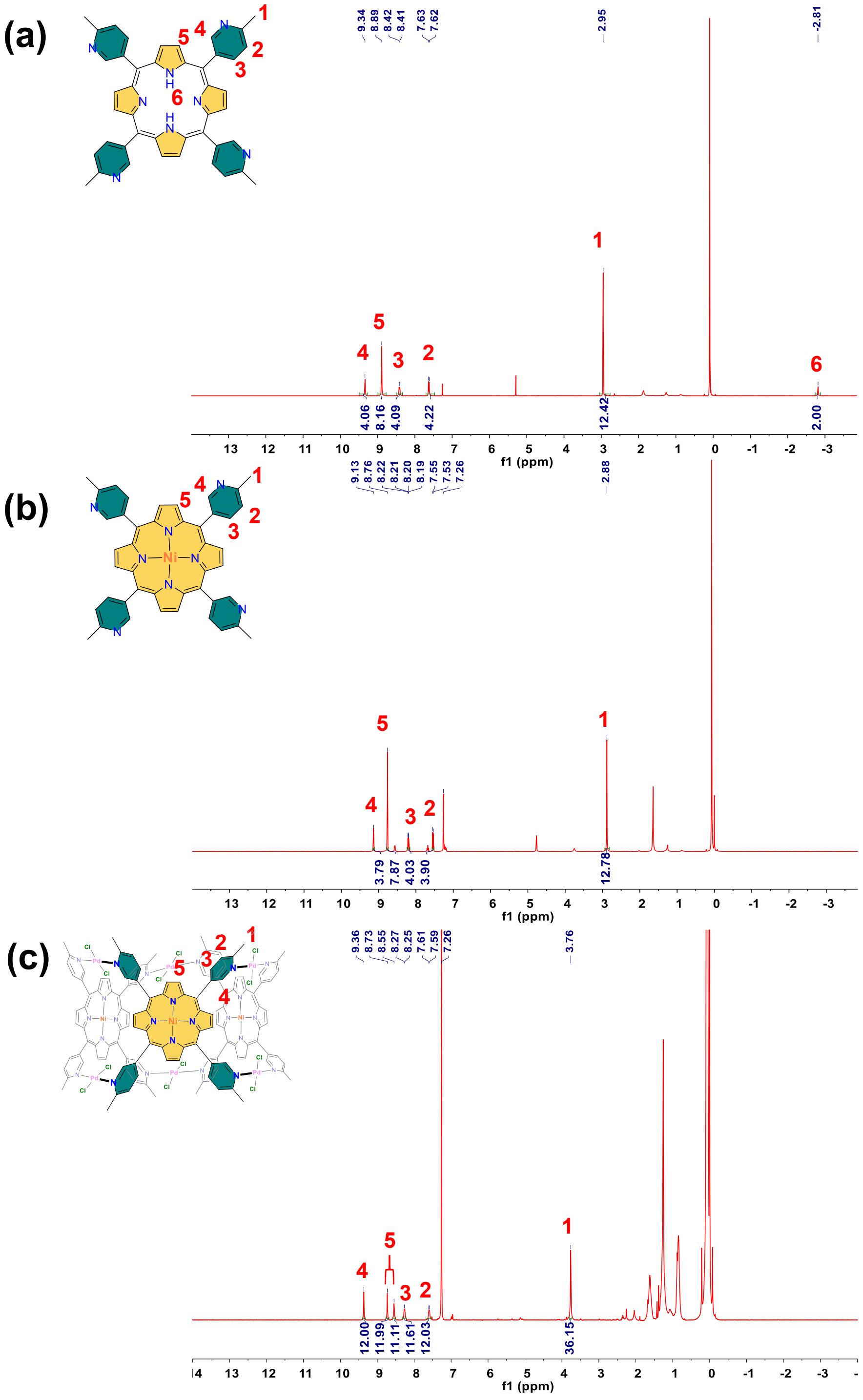

Figure S1 The ^{1}H NMR spectra (400 MHz, 298 K, $CDCl_3$) of **H_2TMPP** (a), TMPP(Ni) (b), and **Pd_6-TMPP(Ni)** (c).

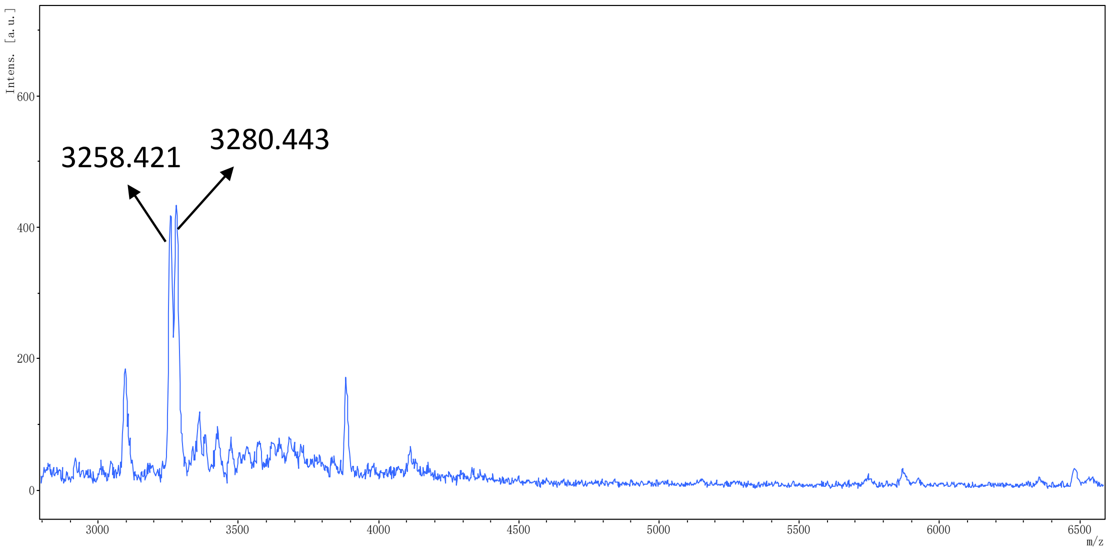

Figure S2 The full MALDI-TOF-MS spectra of **Pd₆-TMPP(Ni)** using DCTB + TFA-Na as the composite matrix.

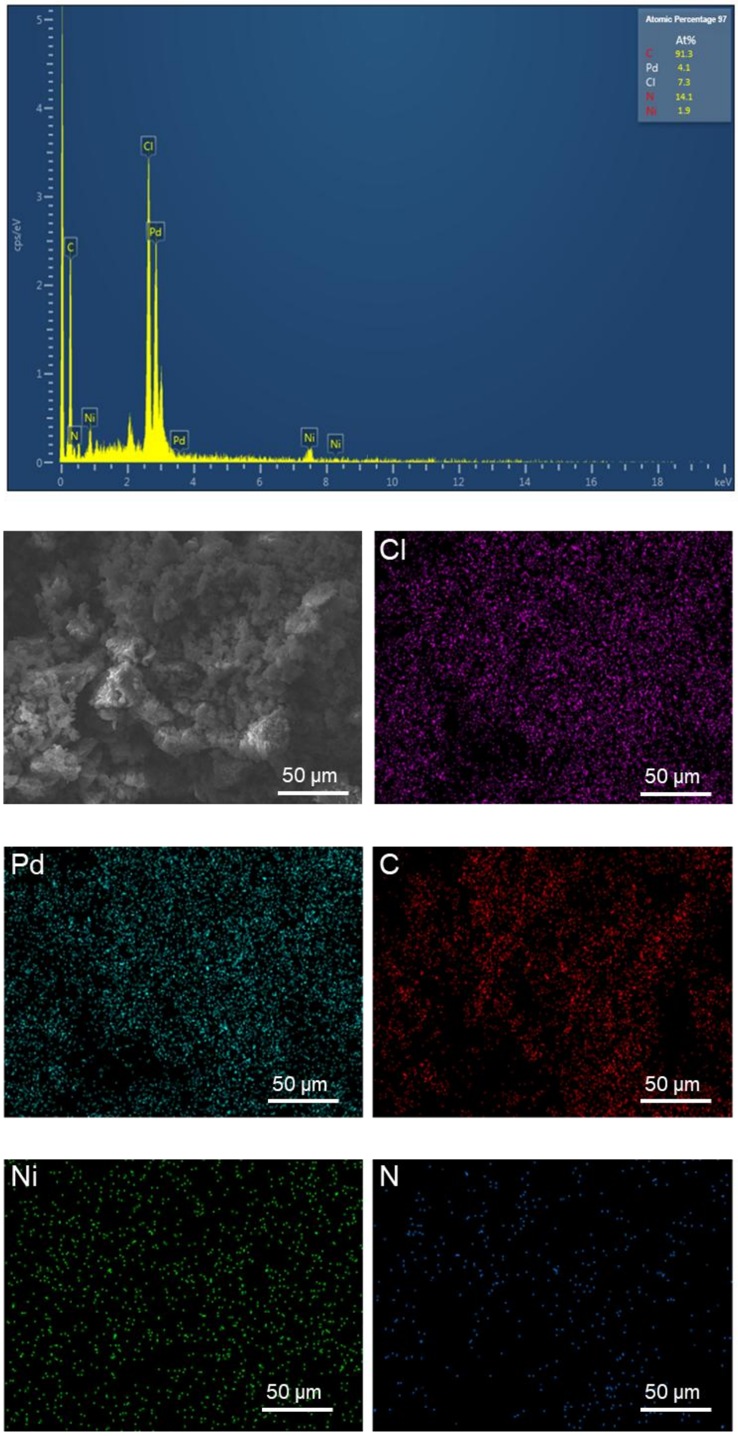

Figure S3 The quantitative elemental ratios of powdery **Pd$_6$-TMPP(Ni)** as characterized by EDS and elemental mapping diagrams showing the average distribution of elements.

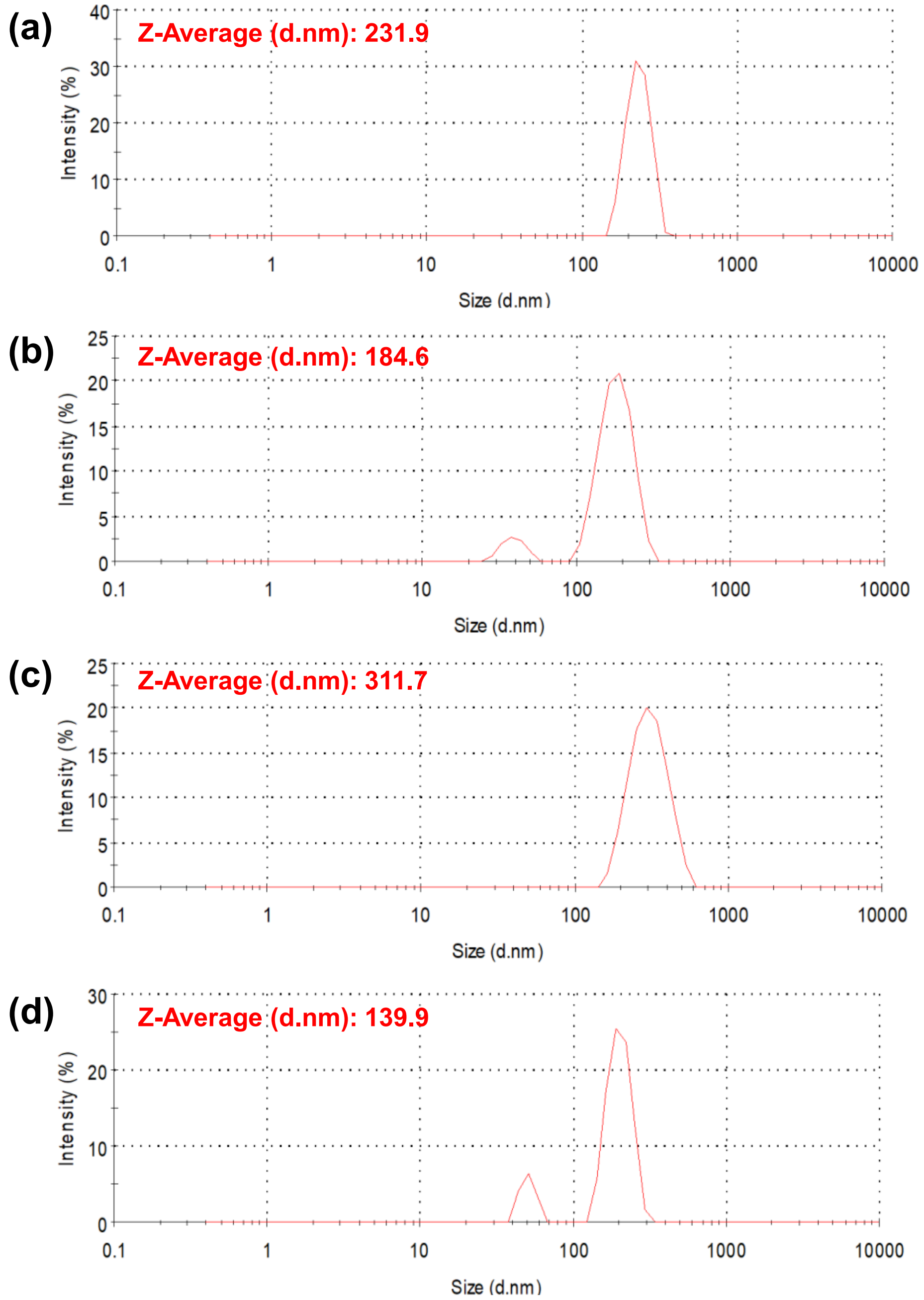

Figure S4 Particle size distribution diagrams of **H_2TMPP-F127** (a), **Pd_6-TMPP(Pd)-F127** (b), **Pd_6-TMPP(Ni)-F127** (c), and blank F127 control (d).

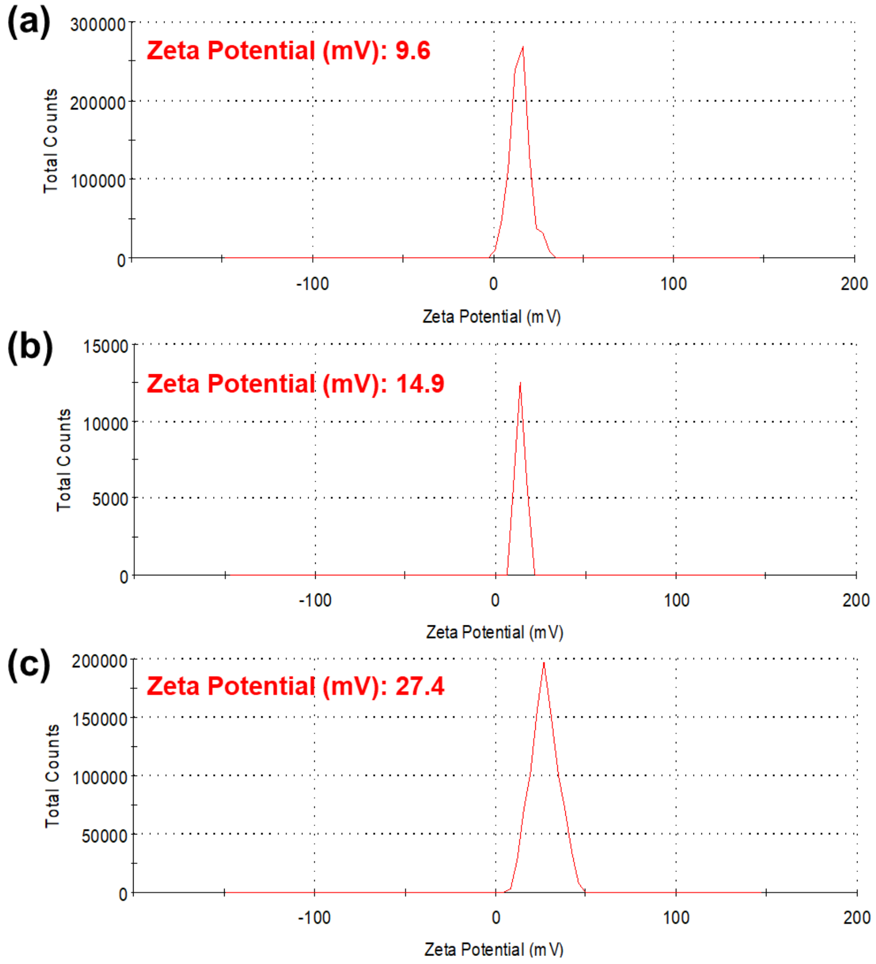

Figure S5 Zeta potential diagrams of **H_2TMPP-F127** (a), **Pd_6-TMPP(Pd)-F127 (b)**, and **Pd_6-TMPP(Ni)-F127** (c).

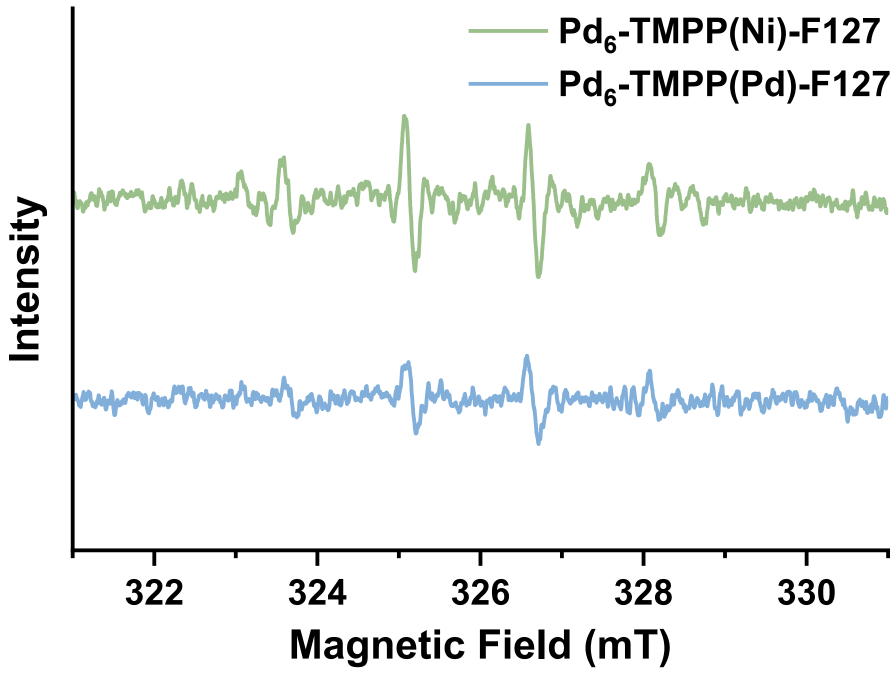

Figure S6 The EPR spectra for •OH as induced by **Pd6-TMPP(Pd)-F127** and **Pd6-TMPP(Ni)-F127**, using DMPO as a spin trap (central magnetic field: 326 mT; tuning frequency: 10 GHz).

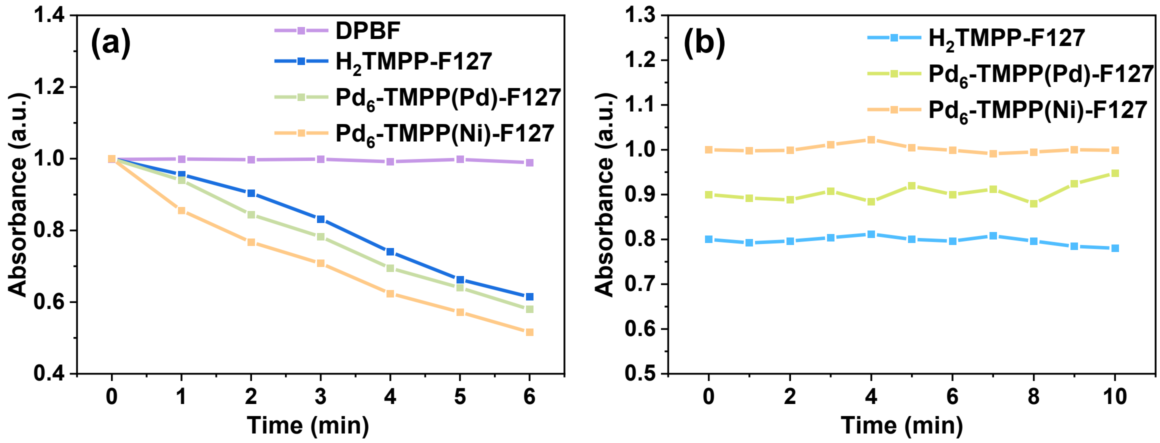

Figure S7 The UV-Vis variation curves showing the differences in $^1O_2$ generation capacity of **H_2TMPP-F127**, **Pd_6-TMPP(Pd)-F127**, and **Pd_6-TMPP(Ni)-F127** compared to blank DPBF control (a). The intensity change in the Soret bands in **H_2TMPP-F127**, **Pd_6-TMPP(Pd)-F127**, and **Pd_6-TMPP(Ni)-F127** under continuous light irradiation (650 nm, 25 mW cm^{-2}) for 10 minutes, showcasing their high photostability (b). For (a), the relative absorbances of the first points were normalized. For (b), the first point of the top line is normalized, and the other two lines are sequentially separated by 0.1 arbitrary units for clarity.

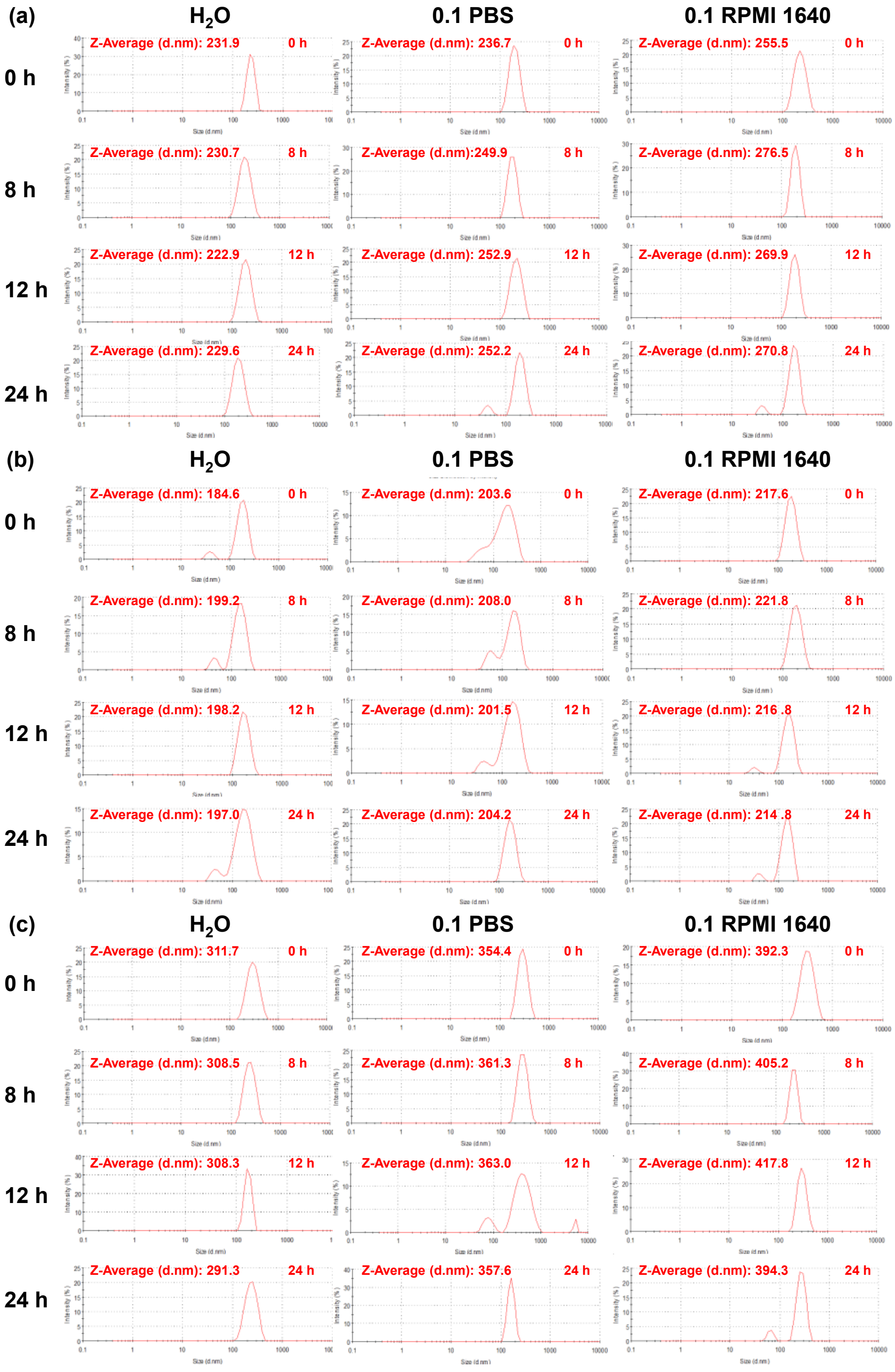

Figure S8 The DLS diagram of **H_2TMPP** (a), **Pd_6-TMPP(Pd)-F127** (b), and **Pd_6-TMPP(Ni)-F127** (c) in H_2O, PBS (0.1×), RPMI 1640 (0.1×) for 0, 8, 12, and 24 h.

2. Supplementary Tables

Table S1 Relevant metal–metal separations (Å), selected bond distances (Å), and bond angles (°) for **Pd_6-TMPP(Ni)**.

Relevant metal–metal separations (Å)			
Pd1···Pd2	12.2020(17)	Pd1···Pd3	13.5157(19)
Pd2···Pd3	11.9926(18)	Pd4···Pd5	12.1185(18)
Pd4···Pd6	13.611(2)	Pd5···Pd6	11.7959(18)
Ni1···Ni2	10.303(3)	Ni1···Ni3	12.463(3)
Ni2···Ni3	10.665(3)		
Selected bond distances (Å)			
Pd(1)-N(1)	2.018(13)	Pd(1)-N(18)	2.023(12)
Pd(1)-Cl(2)	2.292(5)	Pd(1)-Cl(1)	2.304(4)
Pd(2)-N(9)	1.999(12)	Pd(2)-N(2)	2.002(11)
Pd(2)-Cl(4)	2.288(4)	Pd(2)-Cl(3)	2.294(4)
Pd(3)-N(17)	1.985(12)	Pd(3)-N(10)	2.003(13)
Pd(3)-Cl(5)	2.282(5)	Pd(3)-Cl(6)	2.284(5)
Pd(4)-N(20)	1.988(12)	Pd(4)-N(4)	1.994(12)
Pd(4)-Cl(8)	2.276(5)	Pd(4)-Cl(7)	2.291(5)
Pd(5)-N(3)	1.970(10)	Pd(5)-N(11)	2.000(11)
Pd(5)-Cl(9)	2.301(4)	Pd(5)-Cl(10)	2.318(4)
Pd(6)-N(19)	2.011(12)	Pd(6)-N(12)	2.030(13)
Pd(6)-Cl(12)	2.291(5)	Pd(6)-Cl(11)	2.301(5)
Ni(1)-N(6)	1.934(11)	Ni(1)-N(8)	1.939(10)
Ni(1)-N(5)	1.937(11)	Ni(1)-N(7)	1.942(12)
Ni(2)-N(24)	1.960(11)	Ni(2)-N(21)	1.966(11)
Ni(2)-N(22)	1.978(10)	Ni(2)-N(23)	1.983(12)
Ni(3)-N(13)	1.904(12)	Ni(3)-N(15)	1.921(11)
Ni(3)-N(16)	1.939(11)	Ni(3)-N(14)	1.940(12)

Table S1 continued

Selected bond angles (°)

N(1)-Pd(1)-N(18)	172.6(5)	N(1)-Pd(1)-Cl(2)	91.3(4)
N(18)-Pd(1)-Cl(2)	88.9(3)	N(1)-Pd(1)-Cl(1)	89.2(4)
N(18)-Pd(1)-Cl(1)	90.7(3)	Cl(2)-Pd(1)-Cl(1)	178.9(2)
N(9)-Pd(2)-N(2)	178.6(5)	N(9)-Pd(2)-Cl(4)	90.4(4)
N(2)-Pd(2)-Cl(4)	88.3(4)	N(9)-Pd(2)-Cl(3)	91.6(4)
N(2)-Pd(2)-Cl(3)	89.8(4)	Cl(4)-Pd(2)-Cl(3)	176.77(18)
N(17)-Pd(3)-N(10)	172.7(5)	N(17)-Pd(3)-Cl(5)	89.4(4)
N(10)-Pd(3)-Cl(5)	90.2(4)	N(17)-Pd(3)-Cl(6)	90.2(4)
N(10)-Pd(3)-Cl(6)	90.5(4)	Cl(5)-Pd(3)-Cl(6)	177.8(2)
N(20)-Pd(4)-N(4)	172.7(5)	N(20)-Pd(4)-Cl(8)	90.8(4)
N(4)-Pd(4)-Cl(8)	88.9(4)	N(20)-Pd(4)-Cl(7)	88.9(4)
N(4)-Pd(4)-Cl(7)	91.3(4)	Cl(8)-Pd(4)-Cl(7)	179.1(2)
N(3)-Pd(5)-N(11)	178.5(5)	N(3)-Pd(5)-Cl(9)	89.0(4)
N(11)-Pd(5)-Cl(9)	92.0(4)	N(3)-Pd(5)-Cl(10)	89.9(4)
N(11)-Pd(5)-Cl(10)	89.1(4)	Cl(9)-Pd(5)-Cl(10)	178.77(16)
N(19)-Pd(6)-N(12)	173.2(5)	N(19)-Pd(6)-Cl(12)	90.9(4)
N(12)-Pd(6)-Cl(12)	90.9(4)	N(19)-Pd(6)-Cl(11)	88.4(4)
N(12)-Pd(6)-Cl(11)	89.9(4)	Cl(12)-Pd(6)-Cl(11)	179.0(2)
N(6)-Ni(1)-N(8)	171.5(5)	N(6)-Ni(1)-N(5)	89.8(5)
N(8)-Ni(1)-N(5)	89.3(4)	N(6)-Ni(1)-N(7)	90.2(5)
N(8)-Ni(1)-N(7)	91.6(4)	N(5)-Ni(1)-N(7)	174.2(5)
N(24)-Ni(2)-N(21)	90.1(5)	N(24)-Ni(2)-N(22)	179.2(5)
N(21)-Ni(2)-N(22)	89.9(4)	N(24)-Ni(2)-N(23)	89.8(5)
N(21)-Ni(2)-N(23)	174.6(5)	N(22)-Ni(2)-N(23)	90.1(4)
N(13)-Ni(3)-N(15)	172.9(5)	N(13)-Ni(3)-N(16)	89.7(4)

Table S1. continued

N(15)-Ni(3)-N(16)	91.0(4)	N(13)-Ni(3)-N(14)	90.4(5)
N(15)-Ni(3)-N(14)	90.1(5)	N(16)-Ni(3)-N(14)	170.2(5)
N(12)-Pd(6)-Cl(12)	90.9(4)	N(19)-Pd(6)-Cl(11)	88.4(4)
N(12)-Pd(6)-Cl(11)	89.9(4)	Cl(12)-Pd(6)-Cl(11)	179.0(2)
N(6)-Ni(1)-N(8)	171.5(5)	N(6)-Ni(1)-N(5)	89.8(5)
N(8)-Ni(1)-N(5)	89.3(4)	N(6)-Ni(1)-N(7)	90.2(5)
N(8)-Ni(1)-N(7)	91.6(4)	N(5)-Ni(1)-N(7)	174.2(5)
N(24)-Ni(2)-N(21)	90.1(5)	N(24)-Ni(2)-N(22)	179.2(5)
N(21)-Ni(2)-N(22)	89.9(4)	N(24)-Ni(2)-N(23)	89.8(5)
N(21)-Ni(2)-N(23)	174.6(5)	N(22)-Ni(2)-N(23)	90.1(4)
N(13)-Ni(3)-N(15)	172.9(5)	N(13)-Ni(3)-N(16)	89.7(4)
N(15)-Ni(3)-N(16)	91.0(4)	N(13)-Ni(3)-N(14)	90.4(5)
N(15)-Ni(3)-N(14)	90.1(5)	N(16)-Ni(3)-N(14)	170.2(5)

3. References

1. Cao, F.-L.; Zhang, Z.-S.; Dong, M.-L.; Ning, Y.; Zhang, W.-H.; Mao, Y.; Young, D.J. A high-entropy coordination cage featuring an Au-porphyrin metalloligand for the photodynamic therapy of liver cancer. *Chem. Commun.* **2025**, *61*, 6663–6666.
2. Niu, R.-J.; Zhou, W.-F.; Liu, Y.; Yang, J.-Y.; Zhang, W.-H.; Lang, J.-P.; Young, D.J. Morphology-dependent third-order optical nonlinearity of a 2D Co-based metal–organic framework with a porphyrinic skeleton. *Chem. Commun.* **2019**, *55*, 4873–4876.
3. Hu, Q. Nanomaterials of TMPP-based functional coordination complex and their antitumor properties. Soochow University, 2022.
4. Sheldrick, G.M. SADABS (Version 2.03): Program for empirical absorption correction of area detector data; University of Göttingen, Germany. **1996**.
5. Sheldrick, G.M. Crystal structure refinement with SHELXL. *Acta Crystallogr., Sect. C* **2015**, *71*, 3–8.
6. Spek, A.L. PLATON SQUEEZE: A tool for the calculation of the disordered solvent contribution to the calculated structure factors. *Acta Crystallogr., Sect. C* **2015**, *71*, 9–18.
7. Wang, P.; Wang, J.-W.; Zhang, W.-H.; Bai, H.; Tang, G.; Young, D.J. In vitro anticancer activity of nanoformulated mono- and di-nuclear Pt compounds. *Chem. Asian J.* **2021**, *16*, 2993–3000.
